# Glucocorticoid withdrawal and glucocorticoid-induced adrenal insufficiency: Study protocol of the randomized controlled «TOASST” (Taper Or Abrupt Steroid STop) multicenter trial

**DOI:** 10.1371/journal.pone.0281585

**Published:** 2023-04-05

**Authors:** Mathis Komminoth, Marc Y. Donath, Matthias Hepprich, Philipp Schuetz, Claudine A. Blum, Beat Mueller, Jean-Luc Reny, Pauline Gosselin, Gautier Breville, Michael Brändle, Christoph Henzen, Jörg D. Leuppi, Andreas D. Kistler, Robert Thurnheer, Felix Beuschlein, Gottfried Rudofsky, Daniel Aeberli, Peter M. Villiger, Stephan Böhm, Irina Chifu, Martin Fassnacht, Gesine Meyer, Jörg Bojunga, Marco Cattaneo, Constantin Sluka, Helga Schneider, Jonas Rutishauser, for the «TOASST» study group

**Affiliations:** 1 Department of Medicine, Clinical Trial Unit, Cantonal Hospital Baden and University of Basel (J.R.), Basel, Switzerland; 2 Department of Endocrinology, Diabetes and Metabolism, University Hospital Basel, Basel, Switzerland; 3 Department of Medicine, Cantonal Hospital Aarau and University of Basel, Basel, Switzerland; 4 Department of Medicine, University Hospitals Geneva, Geneva, Switzerland; 5 Department of Medicine, Cantonal Hospital St. Gallen, St. Gallen, Switzerland; 6 Department of Medicine, Cantonal Hospital Lucerne, Lucerne, Switzerland; 7 Department of Medicine, Cantonal Hospital Baselland, Liestal, Switzerland; 8 Department of Medicine, Cantonal Hospital Frauenfeld, Frauenfeld, Switzerland; 9 Department of Medicine, Cantonal Hospital Münsterlingen, Münsterlingen, Switzerland; 10 Department of Endocrinology, Diabetology and Clinical Nutrition, University Hospital Zürich and University of Zürich, Zürich, Switzerland; 11 Division of Endocrinology and Diabetes, Cantonal Hospital Olten, Olten, Switzerland; 12 Department of Rheumatology and Immunology, University Hospital Bern, Bern, Switzerland; 13 Medical Center Montbijou, Bern, Switzerland; 14 Department of Medicine, Hospital Bülach, Bülach, Switzerland; 15 Department of Endocrinology and Diabetes, University Hospital Würzburg, Würzburg, Germany; 16 Department of Endocrinology and Diabetes, University Hospital Frankfurt am Main, Frankfurt, Germany; 17 Clinical Trial Unit, University of Basel and University Hospital Basel, Basel, Switzerland; PhD, PLOS, UNITED KINGDOM

## Abstract

**Background:**

Despite the widespread use of glucocorticoids in inflammatory and autoimmune disorders, there is uncertainty about the safe cessation of long-term systemic treatment, as data from prospective trials are largely missing. Due to potential disease relapse or glucocorticoid-induced hypocortisolism, the drug is often tapered to sub-physiological doses rather than stopped when the underlying disease is clinically stable, increasing the cumulative drug exposure. Conversely, the duration of exposure to glucocorticoids should be minimized to lower the risk of side effects.

**Methods:**

We designed a multicenter, randomized, triple-blinded, placebo-controlled trial to test the clinical noninferiority of abrupt glucocorticoid stop compared to tapering after ≥28 treatment days with ≥420 mg cumulative and ≥7.5 mg mean daily prednisone-equivalent dose. 573 adult patients treated systemically for various disorders will be included after their underlying disease has been stabilized. Prednisone in tapering doses or matching placebo is administered over 4 weeks. A 250 mg ACTH-test, the result of which will be revealed *a posteriori*, is performed at study inclusion; all patients are instructed on glucocorticoid stress cover dosing. Follow-up is for 6 months. The composite primary outcome measure is time to hospitalization, death, initiation of unplanned systemic glucocorticoid therapy, or adrenal crisis. Secondary outcomes include the individual components of the primary outcome, cumulative glucocorticoid doses, signs and symptoms of hypocortisolism, and the performance of the ACTH test in predicting the clinical outcome. Cox proportional hazard, linear, and logistic regression models will be used for statistical analysis.

**Conclusion:**

This trial aims to demonstrate the clinical noninferiority and safety of abrupt treatment cessation after ≥28 days of systemic glucocorticoid therapy in patients with stabilized underlying disease.

**Trial registration:**

ClinicalTrials.gov Identifier: NCT03153527; EUDRA-CT: 2020–005601–48 https://clinicaltrials.gov/ct2/show/NCT03153527?term=NCT03153527&draw=2&rank=1.

## Introduction

### Background and rationale

Glucocorticoids have been in clinical use since 1948 [1] and remain among the most widely used medications worldwide today [2]. They are used primarily to treat inflammatory and autoimmune diseases. Among the numerous and well-known untoward drug effects, the risk for skeletal, metabolic, dermatologic, and ophthalmologic treatment complications increases with treatment duration and dose [2,3]. Therefore, therapy should be restricted to the minimal time necessary to control the underlying disease [4,5]. On the other hand, premature termination may provoke relapses, and abrupt glucocorticoid withdrawal can result in iatrogenic hypocortisolism or even adrenal crisis [6–9]. In this therapeutic dilemma, various tapering regimens are used, which differ greatly among disorders and physicians and are often based on expert opinions rather than solid evidence, as there are no studies proving the superiority of one regime over the others [10–12]. To complicate matters further, signs and symptoms of glucocorticoid-induced hypocortisolism are nonspecific [13], and whether the risk of glucocorticoid-induced suppression of the hypothalamic-pituitary-adrenal axis correlates with treatment duration and/or cumulative dose is controversial [6,8,14–16], although such a correlation is often intuitively assumed. Therefore, despite some limited evidence that abrupt stopping is safe if patients are instructed on glucocorticoid stress cover dosing [17], ACTH stimulation tests are used in clinical routine to evaluate the adrenal reserve and decide on the necessity of stress cover doses [18,19]. However, test protocols are heterogeneous, and the tests’ power to guide glucocorticoid prescription practice or predict clinical outcome has not been prospectively evaluated.

Thus, despite more than seven decades of clinical experience with systemically administered glucocorticoids, crucial knowledge on their use is still lacking, particularly regarding the termination after prolonged systemic treatment [20]. Based on these shortcomings and own observations on adrenal function in participants of the “REDUCE” trial [13], we hypothesize that it is feasible and safe to rapidly terminate glucocorticoid therapy, once the underlying disease has been adequately controlled and provided glucocorticoid cover is ensured in situations of stress.

To test this hypothesis, we designed a randomized controlled trial powered to prove clinical noninferiority of abrupt treatment stop compared to four weeks of tapering after 28 days of therapy, irrespective of the functional status of the adrenal glands as assessed by the 250 mcg adrenocorticotropic hormone (ACTH) test.

### Trial design and methods

The “TOASST” (Taper Or Abrupt Steroid STop) trial is an investigator-initiated, randomized, triple-blinded (patients, care providers, investigators), placebo-controlled, multi-center, noninferiority trial, comparing rapid termination of systemic glucocorticoid treatment with a tapering regime over 4 weeks. The trial has been registered (ClinicalTrials.gov Identifier: NCT03153527; EUDRA-CT: 2020-005601-48).

### Ethical considerations

The study protocol was approved on July 13, 2016 by the competent ethics committee Ethikkommission Nordwest- und Zentralschweiz (EKNZ; BASEC approval number 2016–00487). The trial is performed in accordance with the current version of the Declaration of Helsinki and the guidelines of Good Clinical Practice issued by International Conference on Harmonization of Technical Requirements for Registration of Pharmaceuticals for Human Use.

Placebo as comparator is ethically justifiable. At the time of inclusion, the underlying disease will have been stabilized by ≥28 days of treatment with systemic glucocorticoids alone or in combination with other immunosuppressants. At this time, the glucocorticoid has usually been reduced gradually to a dose of 7.5 mg prednisone-equivalent (i.e. the “Cushing threshold”) or slightly above. Although glucocorticoid withdrawal carries a risk of provoking adrenal crisis, our own data suggest that in patients instructed carefully about stress-cover dosing, the danger of clinically relevant secondary adrenal failure is relatively small [13,17]. All trial participants will be supplied with appropriate information and prednisone tablets for situations with increased needs for glucocorticoids.

## Material and methods

### Participating sites and competent ethic committees

The following sites participate in the study. Ethical approval has been obtained for each site by the respective competent ethics committee (listed in parentheses).

Cantonal Hospital Baden, 5404 Baden, Switzerland (Ethics Commission Northwest- und Central Switzerland EKNZ)

University Hospital Basel, 4031 Basel, Switzerland (Ethics Commission Northwest- und Central Switzerland EKNZ)

Cantonal Hospital Aarau, 5001 Aarau, Switzerland (Ethics Commission Northwest- und Central Switzerland EKNZ)

University Hospital Geneva, 1205 Geneva, Switzerland (Ethics Committee Geneva)

Cantonal Hospital St. Gallen, 9000 St. Gallen, Switzerland (Ethics Committee Eastern Switzerland EKOS)

Cantonal Hospital Baselland, Liestal, Switzerland (Ethics Commission Northwest- und Central Switzerland EKNZ)

Cantonal Hospital Frauenfeld, 8501 Frauenfeld, Switzerland (Ethics Committee Eastern Switzerland EKOS)

Cantonal Hospital Münsterlingen, 8696 Münsterlingen, Switzerland (Ethics Committee Eastern Switzerland EKOS)

University Hospital Zürich, 8091 Zürich, Switzerland (Ethics Committee Zürich)

Cantonal Hospital Olten, 4600 Olten, Switzerland (Ethics Commission Northwest- und Central Switzerland EKNZ)

University Hospital Bern, 3010 Bern, Switzerland (Cantonal Ethics Committee Bern)

Hospital Bülach, 8180 Bülach, Switzerland (Ethics Committee Zürich)

University Hospital Würzburg, 97080 Würzburg, Germany (Ethics Committee of the University of Würzburg)

University Hospital Frankfurt, 60590 Frankfurt, Germany (Ethics Committee of the Johann Wolfgang Goethe University, Frankfurt)

### Participants

Eligible patients have been under systemic glucocorticoid treatment for autoimmune or inflammatory gastrointestinal, rheumatologic, pulmonary, haemato-oncological, endocrine, or other disorders. The treating physician decides whether the activity of the underlying disorder permits a potentially abrupt treatment stop. At the time of inclusion, patients must be on a daily dose of at least 7.5 mg prednisone equivalent, i.e. high enough to still potentially suppress the HPA axis.

### Inclusion criteria

Participants fulfilling all of the following inclusion criteria are eligible for the study:

- Informed Consent as documented by signature
- Age ≥ 18 years
- Daily glucocorticoid dose ≥ 7.5 mg prednisone-equivalent at the time of inclusion
- Therapy over ≥ 28 days, with ≥ 7.5 mg prednisone-equivalent average daily dose and ≥ 420 mg prednisone-equivalent cumulative glucocorticoid dose prior to inclusion
- Tapering not or no longer mandatory to treat underlying disease

### Exclusion criteria

Patients fulfilling one or more of the following criteria will not be included into the study:

- Primary adrenal failure
- Treatment with systemic depot glucocorticoids (e.g. intramuscular, epidural)
- Incapability to administer glucocorticoid cover treatment in situations of stress
- Inability or unwillingness to provide informed consent
- Women who are pregnant or breast feeding,
- Intention to become pregnant during the study,
- Lack of safe contraception, defined as female participants of childbearing potential, not using and not willing to continue using a medically reliable method of contraception for the entire study duration, such as oral, injectable, or implantable contraceptives, or intrauterine contraceptive devices, or who are not using any other method considered sufficiently reliable by the investigator in individual cases.
- Known or suspected non-compliance
- Inability to follow the procedures of the study, e.g. due to language problems, psychological disorders, dementia
- Participation in another study with investigational drug within 30 days preceding and during the present study
- Previous enrolment into this study
- Enrolment of the investigator, his/her family members, employees and other dependent persons

### Outcomes

**Fig 1:**
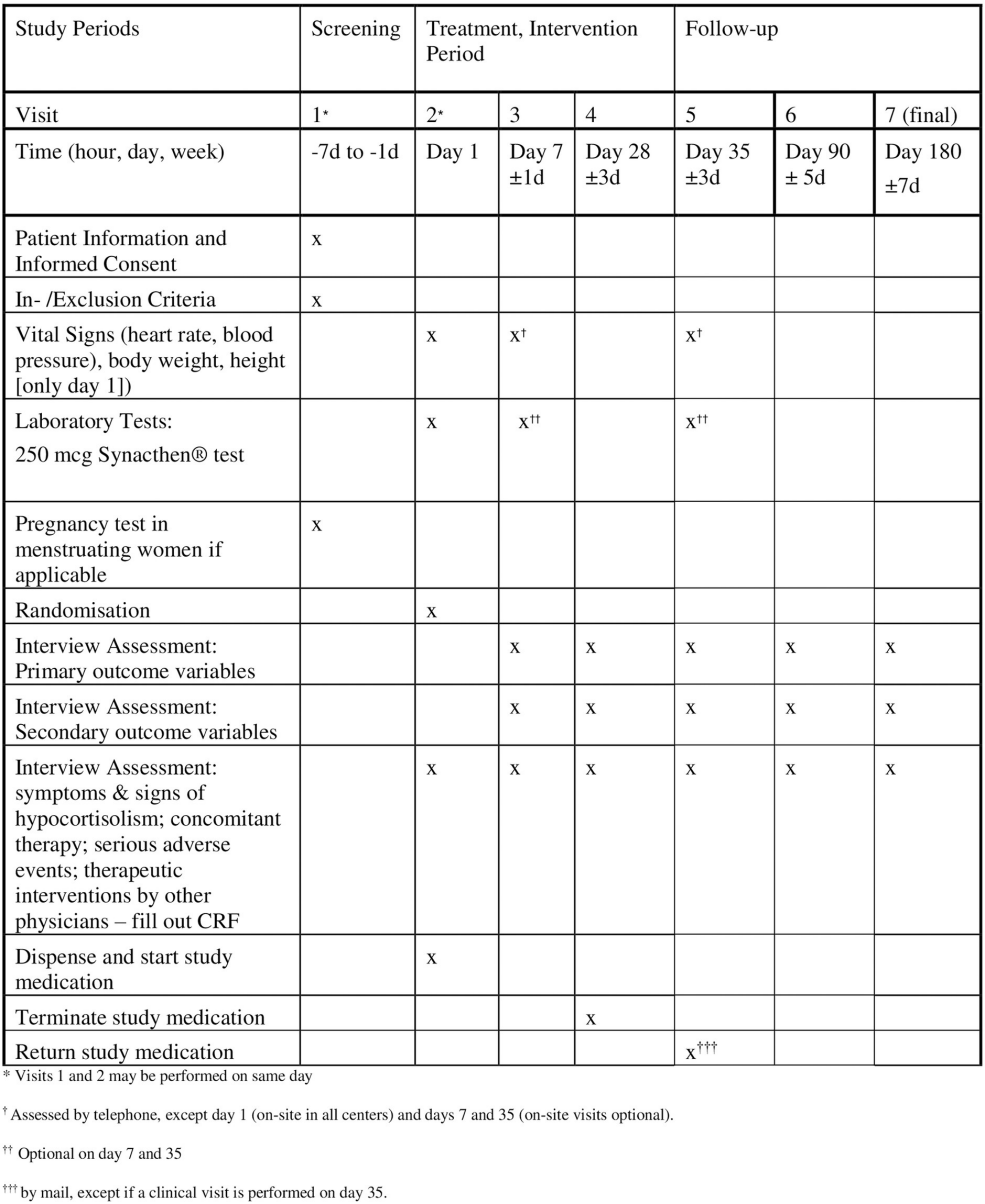
Study schedule.

**Fig 2:**
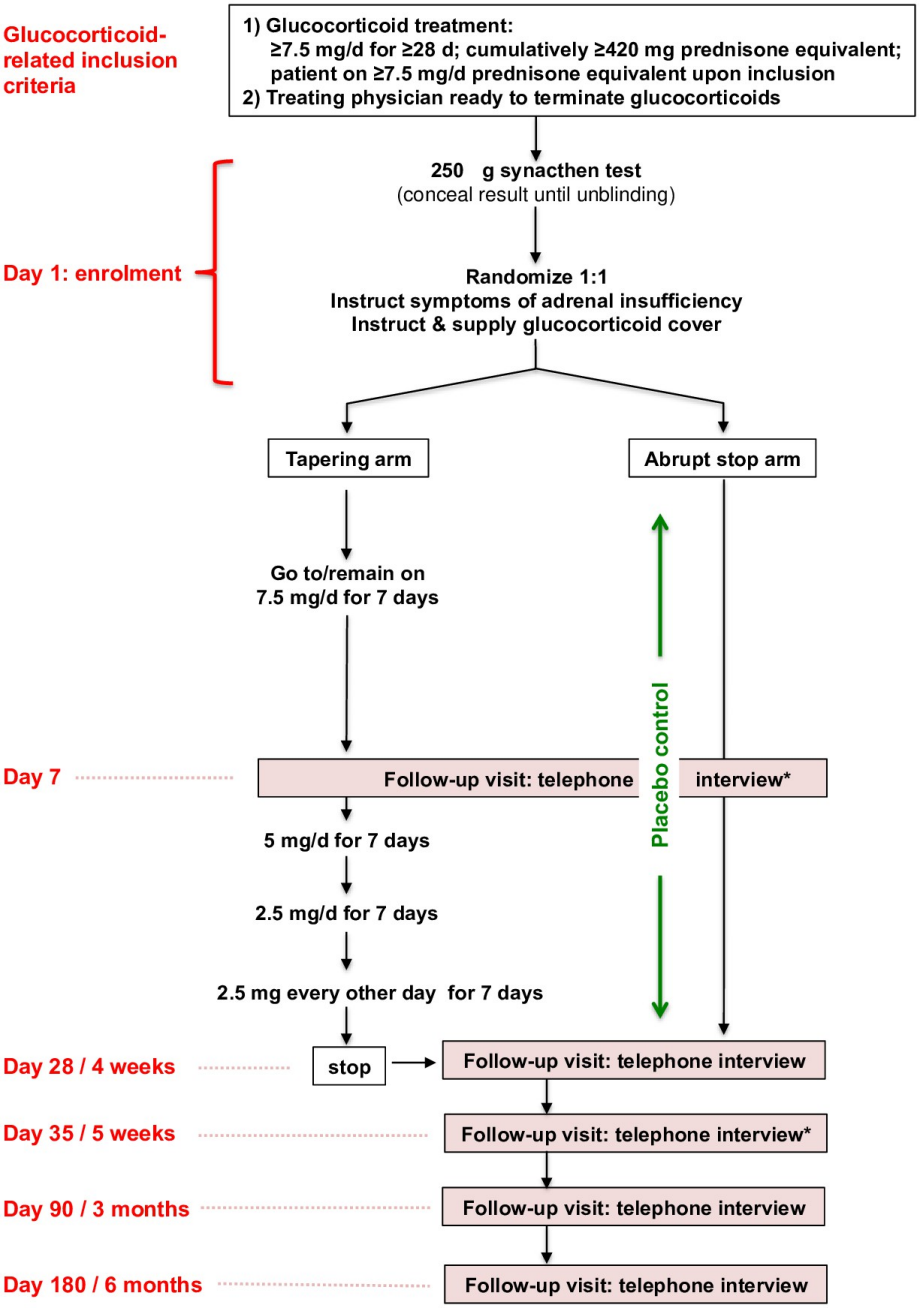
Study flow. *Days 7 and 35: optional on-site visits to assess vital signs and perform 250 mcg Synacthen^®^ stimulation test.

Outcome measures are assessed at 5 telephone interviews over a follow-up period of 6 months (Figs 1 and 2). Optionally, two follow-up visits (days 7 and 35) are performed at the trial site.

#### Primary outcome measure

This is a composite endpoint with the following components: Time to first occurrence of hospitalization, death, initiation of unplanned systemic glucocorticoid therapy, or adrenal crisis (defined as glucocorticoid-responsive hypotension or shock with or without accompanying symptoms and signs such as weakness, apathy, nausea, vomiting, abdominal pain, hypothermia, hyponatremia [serum sodium < 135 mM], hyperkalemia [serum potassium > 5 mM], hypoglycemia [plasma glucose < 3.5 mM]); whichever occurs first.

### Secondary outcome measures

1. Time to first occurrence of individual components of the primary outcome
2. Total cumulative systemic glucocorticoid dose
3. Cumulative systemic glucocorticoid dose administered to treat or prevent adrenal failure
4. Cumulative systemic glucocorticoid dose administered to treat relapse of disease, specified for each disease
5. General health status as self-assessed by the participant on a visual analog scale (VAS) from 0 to 100, assessed at days 1, 7, 28, 35, 90, 180
6. Score of symptoms and signs of hypocortisolism: weakness, hypothermia, nausea, vomiting, abdominal pain, fatigue, dizziness. Blood pressure (supine and standing) is assessed upon enrolment in all participants and optionally at clinical visits on day 7 and 35.
7. Performance in the 250 mcg ACTH (Synacthen^®^) test: test performed upon enrolment in all participants and optionally on days 7 and 35 and during clinical visits
8. In patients hospitalized upon enrolment: length of hospital stay.

### Intervention, examinations and follow-up

Fig 1 shows the study schedule, and Fig 2 shows the study flow.

In total, 573 patients will be enrolled. Patients are randomly assigned in a 1:1 ratio to either prednisone in decreasing doses over 4 weeks or placebo. Patients, treating physicians, and study personnel are blinded to treatment allocation. Given the absence of standardized tapering regimens, the planning committee settled on a dose-reduction plan over four weeks, starting at 7.5 mg of prednisone daily. At trial entry, vital signs, including heart rate and blood pressure in recumbent and upright positions are recorded, and a 250 mcg Synacthen^®^ stimulation test is performed. Treating physicians and investigators are unaware of the results, and the performance of the test in predicting the clinical outcome will only be assessed after completion of the trial. As a safety measure, all patients are instructed about signs and symptoms of hypocortisolism as well as glucocorticoid stress coverage and are provided with emergency prednisone medication.

Serum and buffy coat samples are collected upon enrolment and at on-site visits and stored in a biobank, provided specific consent is obtained from the participant.

Treating physicians are informed about their patients’ participation in the trial to increase awareness of potentially occurring hypoadrenergic symptoms. Prescription of any additional medications, including other immunosuppressants and the re-start of glucocorticoid therapy, is entirely at the discretion of the treating physician and/or the patient.

At all follow-up visits, primary and secondary outcome measures are ascertained by telephone interview, and data are entered into the electronic case report form (eCRF) on the data capture platform secuTrial^®^, which runs on a secured server hosted by the University Hospital Basel. Optionally, visits on day 7 and 35 are performed at the trial site in order to perform Synacthen^®^ tests and monitor vital signs.

### Randomization and treatment allocation

The randomization list was generated by the Clinical Trial Unit, University Hospital, Basel. Patients are randomized online using the secuTrial^®^ platform in a 1:1 ratio, stratified by trial site, age group, and daily prednisone dose at inclusion, to either prednisone in decreasing doses over 4 weeks or matching placebo. To avoid predictable alternation of treatment allocation, and thus potential loss of allocation concealment, patients are allocated with a probability of 70 percent to the treatment group that would minimize the difference between the groups within the patient’s stratum.

### Blinding and unblinding

5 mg Prednisone tablets and matching placebo are packaged into generically labeled glass vials. Trial participants, treating physicians and study staff are unaware of treatment allocation. Unblinding is permissible if knowledge of a patient’s treatment allocation is essential for further therapy. Unblinding must be performed in accordance with the local principal investigator or the trial’s sponsor/investigator and is executed via the online database tool (SecuTrial^®^).

### Safety considerations

The individual components of the primary outcome are defined as safety outcomes. The occurrence of an adrenal crisis is of particular relevance due to its obvious danger for the patient. A data and safety monitoring board (DSMB), consisting of three clinical experts not involved in the conduct of the study and an independent statistician, convenes after the first 100 patients are included in the trial and yearly thereafter.

Two types of safety analyses are performed: periodic analyses for the DSMB’s meetings, and a final safety analysis at the end of the trial. Safety analyses for DSMB meetings will summarize the rates of safety outcomes pooled over both treatment arms. The DSMB may advise early termination of the trial on the basis of participants’ safety concerns.

The final safety analysis for the trial will summarize the rates of safety outcomes stratified by treatment arm and will be performed by the trial statistician after study completion.

### Adverse event reporting

A total of 11 potential glucocorticoid-associated side effects are monitored as adverse events (AE; Table 1).

**Table 1:** Glucocorticoid-associated side effects documented as adverse events (AE)*.

Infection; self-reported or documented in patient’s medical record
Allergic reaction; self-reported or documented in patient’s medical record
Arterial hypertension; defined as new medication or increase of dose of present medication as prescribed by treating physician
Hyperglycemia or diabetes mellitus; defined as new medication or increase of dose of present medication as prescribed by treating physician
Psychological symptoms, i.e. mood swings, depression, or hallucinations; either self-reported or newly diagnosed or evidenced by increase of dose of present medication by treating physician)
Gastrointestinal symptoms, defined as nausea, heartburn, or documented ulcer disease
Osteoporosis, as evidenced by osteodensitometry scan result or start of therapy by treating physician
Aseptic bone necrosis, as documented in patient’s medical record
Increase in weight; defined as self-reported by patient or documented by treating physician or study team at on-site visit
Skin alterations; defined as thinning of skin, redness of skin, easy bruising, bleeding; as self-reported by patient or documented by treating physician or study team at on-site visit)
Muscle weakness; defined as self-reported decrease in muscular strength

*AEs correspond to side-effects as listed for systemic glucocorticoid preparations and published by the Swiss Agency for Therapeutic Products; www.swissmedicinfo.ch.

Serious adverse events (SAE) are defined as death, life-threatening disease, hospitalization (except if electively planned prior to trial inclusion), persistent disability, or other medically important conditions. AE, SAE, and suspected unexpected serious adverse drug reactions (SUSARs) are reported via the eCRF in secuTrial^®^ to the Sponsor-Investigator of the study within 24 hours after their assessment. Notification of competent authorities is done according to pertinent legislation.

An annual safety report is submitted once a year to the competent ethics committees and authorities.

### Monitoring

An audit trail system maintains a record of all entries into the secuTrial^®^ data base. Monitoring is conducted according to the trial’s monitoring plan. In brief, on-site monitoring visits are performed after enrolment of the first 2 to 4 patients, followed by telephone visits 6 to 12 months thereafter. In addition, a remote online monitoring of the secuTrial^®^ data base is performed monthly. Further on-site visits are planned according to individual trial sites’ demand. A close-out visit will be performed after the last patient’s last visit.

### Statistical considerations

#### Hypothesis

The aim of the trial is to test the hypothesis that rapid termination of glucocorticoid treatment (experimental arm) is noninferior to the tapering regime (control arm) with regard to the time to event (primary composite outcome). Accordingly, the null hypothesis corresponds to inferiority of the abrupt glucocorticoid tapering regime.

#### Sample size

To assess the primary outcome measure, a Cox proportional hazards regression model will be fitted to the data. Noninferiority will be concluded if the two-sided 95% confidence interval of the hazard ratio between the experimental and the control arm lies entirely below the critical hazard ratio defined as

$$\text{HR}=\frac{\lambda_{\text{e}}}{\lambda_{\text{e}}}=\frac{\frac{-\text{log}(\pi_{\text{et}})}{\text{t}}}{\frac{-\text{log}(\pi_{\text{ct}})}{\text{t}}}=\frac{-\text{log}(\pi_{{\text{e}}_{\text{t}}})}{-\text{log}(\pi_{{\text{c}}_{\text{t}}})}=\frac{-\text{log}(\pi_{{\text{c}}_{\text{t}}}-\text{m})}{-\text{log}(\pi_{{\text{c}}_{\text{t}}})}$$

where λ_e_ and λ_c_ are the hazard rates in the experimental and control arms, t is a fixed point of time, π_et_ and π_ct_ are the proportions of event-free patients at time t, and m is the noninferiority margin expressed as the additional proportion of patients having had an event in the experimental arm, assuming that the occurrence of events follows an exponential distribution. Noninferiority will be concluded if the upper limit of the two-sided 95% confidence interval of the hazard ratio lies below the critical hazard ratio.

For the sample size estimation, the following assumptions were made:

- Patients are randomized to the control and experimental arms in a 1:1 ratio.
- After inclusion, all patients are followed for 6 months. Patients without an event within 6 months are considered right-censored and are not followed any further even though the trial might be ongoing.
- 10% of patients will drop out within the 6 months follow-up period. The drop-out events are distributed uniformly over this period.
- The event rate within the 6 months follow-up period, i.e., the proportion of patients with an event, is 40%, both for the control and the experimental arms. We estimated this overall figure based on expected relapse rates for the various eligible diseases, as indicated by clinical experts and published data, e.g. in COPD or inflammatory bowel disease. Our estimation is conservative since the other components of the combined primary outcome will contribute to the event rate.
- The survival curves in the control and experimental arms follow the same exponential distribution. The rate parameter of the exponential distribution is chosen so that the corresponding cumulative distribution function value at 6 months equals the assumed event rate.
- The noninferiority margin is defined as an absolute increase of the event rate by 13% over 6 months. We used a modified Delphi approach to determine this margin. 10 experienced clinicians board-certified in internal medicine and/or rheumatology, gastroenterology, and endocrinology were individually asked to define the maximum increase in outcome events they would accept for the benefit of rapid glucocorticoid stop and gain of high-quality scientific data in the area of steroid withdrawal. Assuming an event rate of 40% in the standard treatment arm, the mean tolerable increase under experimental treatment was 13.6% (range, 5 to 20%; median, 13%).
- The significance level is 5%.

For each combination of the noninferiority margins 7.5%, 7.6%, …, 17.5%, and the sample sizes n = 200, 210, …, 1200, 999 individual data sets were simulated based on the above assumptions. A Cox proportional hazards regression model was fitted to each data set, and the hazard ratios for the experimental *vs*. the control arm with the associated 95% confidence interval were estimated. Noninferiority was concluded if the upper limit of the two-sided 95% confidence interval of the hazard ratio was below the critical hazard ratio (see above equation). The power was estimated as the proportion of positive conclusions divided by the total number of the 999 iterations per parameter combination. To achieve a power of 80%, 573 patients (95% CI, 565 to 580) need to be included, assuming an event rate of 40% over the 6 months follow-up period and setting the noninferiority margin to 13%. The critical hazard ratio for this noninferiority scenario is 1.478.

The sample size of n = 573 is a conservative estimation, since the assumed drop-out rate of 10% is relatively high.

### Planned analyses

Detailed methodology for summaries and statistical analyses of the data collected in this trial will be documented in a statistical analysis plan. The statistical analysis plan will be finalized before database closure and will be under version control at the Clinical Trial Unit, University Hospital Basel. Deviations from the analyses described below will be listed in a separate section of the analysis plan.

#### Datasets to be analyzed, analysis populations

The full analysis set (FAS) will include all patients who were randomized, gave written informed consent, and started the treatment. The per protocol set (PPS) will include all patients in the FAS who fulfilled the eligibility criteria and for whom the treatment was completed as planned in the study protocol.

All statistical analyses will be performed on the FAS according to the intention-to-treat principle (i.e. all patients will be analyzed on the basis of the treatment to which they were randomly allocated), except for the primary, non-inferiority analysis performed on the PPS (with patients analyzed on the basis of the treatment actually received).

#### Primary analysis

A Cox proportional hazards regression model with time to the primary endpoint as the response and treatment arm as the explanatory variable will be fitted, stratified according to study center, daily prednisone dose at inclusiuon, and age group. Noninferiority will be concluded if the two-sided 95% confidence interval of the hazard ratio (HR) between the experimental and the control arm lies entirely below the critical HR ratio of 1.478. The analysis will be conducted on the PP set.

#### Secondary analyses

The secondary outcomes will be analyzed as follows.

Performance of the 250 mcg corticotropin stimulation test: If this test is clinically relevant, it is assumed that in patients with a normal test result, the two study arms will not differ with regard to the primary outcome, whereas patients with a pathological test result will experience an event more often after the rapid termination (experimental arm) than after tapering (control arm). It is thus hypothesized that there is an interaction between the test result and the glucocorticoid withdrawal scheme. To test for a significant interaction, the Cox proportional hazard regression model used to assess the primary objective is extended by the stimulation test result (passed vs. failed) and its interaction with the treatment group (rapid vs. tapering). If a significant interaction is found, subgroup analyses are performed for the two treatment groups in order to estimate the strength of the association between test result and primary outcome.

The time to first occurrence of individual components of the primary outcome is, in analogy to the composite primary outcome, also analyzed with Cox proportional hazards regression models.

Cumulative overall systemic glucocorticoid dose, cumulative systemic glucocorticoid dose administered to treat or prevent adrenal failure, cumulative systemic glucocorticoid dose administered to treat relapse of disease (specified for each disease), general health status as self-assessed by the patients on a VAS, blood pressure (supine and standing), hospital length of stay, weakness, hypothermia, nausea, vomiting, abdominal pain, and fatigue are analyzed with linear regression models.

In all analyses, the variables for which randomization was stratified (center and age group) are included as covariates for adjustment. If model assumptions are not met, transformations of outcome variables and the use of generalized linear (instead of linear) models are considered.

Potential glucocorticoid-induced side effects will be assessed and documented on the CRF. Proportions of side effects will be compared between treatment groups and analyzed using logistic regression.

#### Subgroup analyses

Pre-specified subgroups defined by the following parameters will be analyzed by the trial statistician:

1. Daily glucocorticoid dose (in mg prednisone-equivalent) at the time of inclusion
2. Cumulative glucocorticoid dose (in mg prednisone-equivalent) in the 28 days prior to inclusion
3. Sex
4. Concomitant immunosuppressive drugs at inclusion or during follow-up
5. Underlying disorder
6. Result of the 250 mcg synacthen^®^ test

#### Interim analyses

Once 70% of the planned sample size will have their primary endpoint assessed, we will perform a blinded, pooled, sample size re-estimation. To this end, the global rate of events—pooled over both treatment arms—will be calculated and compared to the assumed rate in the sample size estimation. The sample size estimation will be repeated as performed originally and the study’s sample size adjusted accordingly in case more patients are needed. In no case will the originally calculated sample size be reduced.

#### Safety analysis

Two types of safety analyses will be performed: periodic analyses for the DSMB’s meetings, and a final safety analysis at the end of the trial.

Safety analyses for DSMB meetings will summarize the rates of safety outcomes (as defined above, section 10) pooled over both treatment arms. Event types and relevance as defined for SAEs will be included in the report. The DSMB has the right to request an analysis stratified by treatment arm, or completely unblinded in case of concern for patient safety. Safety analysis for the DSMB is performed by the independent DSMB statistician.

The final safety analysis for the trial will summarize the rates of safety outcomes stratified by treatment arm and will be performed by the trial statistician after study completion.

### Deviation(s) from the original statistical plan

If substantial deviations of the analyses as outlined in these sections are needed for whatever reason, the protocol will be amended. All deviations from the protocol or from the detailed statistical analysis plan will be listed and justified in a separate section of the final statistical report.

### Handling of missing data and drop-outs

We will strive to make a complete case analysis, which will likely be feasible because the primary outcome is composed of clearly defined clinical events that can easily be tracked and inquired from the treating physicians if necessary. If a substantial proportion of patients have missing data, we will consider the use of multiple imputation.

Patients who leave the trial during the blinded treatment phase are replaced only if the number of total dropouts is >10%.

## Discussion

This investigator- initiated, multicenter, randomized controlled trial aims to establish the noninferiority of an abrupt glucocorticoid treatment stop after a minimum of 28 days of systemic therapy for an inflammatory or autoimmune disorder. Study inclusion requires a cumulative dose of at least 420 mg prednisone-equivalents with a mean daily dose of at least 7.5 mg, and a daily dose of ≥7.5 mg prednisone equivalent upon enrolment. Prior to inclusion, most patients will have tapered the glucocorticoid dose according to the course of the underlying disease; thus, we expect the majority of patients to be on 7.5 to 15 mg by the time patients are considered eligible for participation by their treating physicians.

The “TOASST” trial will provide high-quality evidence about the unanswered question of how to terminate prolonged glucocorticoid treatment, once the underlying disease has been stabilized. A randomized controlled trial over 24 weeks has shown clinical benefit of continued low-dose prednisone treatment over tapering in patients on tocilizumab therapy for rheumatoid arthritis [21], but there were no clinically meaningful between-group differences in observed serious adverse events, and there was no comparison between tapering and abrupt treatment stop. Data from prospective studies evaluating stopping glucocorticoid treatment termination are scarce for most inflammatory and autoimmune disorders, and tapering or stopping strategies are heterogeneous rather than evidence-based. Our trial will also evaluate prospectively the performance of the 250 mcg ACTH test in predicting the need for unplanned glucocorticoid therapy and the clinical outcome after ending prolonged treatment. To our knowledge, this has not been done in a trial of comparable size.

Limitations include the heterogeneity of the disorders included in the trial and, consequently, the relatively small number of participants with a given disorder. Also, the tapering regimen in the “standard” treatment arm was determined according to a consensus among the members of the planning committee, rather than following a generally accepted treatment plan, since evidence-based data on how to taper glucocorticoids are lacking.

In conclusion, the”TOASST” trial addresses the question of how to terminate prolonged glucocorticoid treatment, providing high-quality evidence to a relevant aspect that has remained unresolved more than 70 years after glucocorticoids were introduced in clinical practice.

## Supporting information

S1 File SPIRIT checklist with administrative data. (PDF)

S2 File Trial protocol V3.0 (Switzerland). (PDF)

S3 File Trial protocol V3.1 (Germany). (PDF)

## Abbreviations

ACTH: Adrenocorticotropic hormone
AE: Adverse Event
CRF: Case Report Form
CTU: Clinical Trials Unit
DSMB: Data and Safety Monitoring Board
eCRF: Electronic Case Report Form
FAS: Full Analysis Set
HPA: Hypothalamic-Pituitary-Adrenal
ITT: Intention to treat
PP: Per Protocol
SAE: Serious Adverse Event
SUSAR: Suspected Unexpected Serious Adverse Drug Reaction
