# Supplementary material for: Glucocorticoid withdrawal and glucocorticoid-induced adrenal insufficiency: Study protocol of the randomized controlled «TOASST” (Taper Or Abrupt Steroid STop) multicenter trial
Source: PLoS One. 2023 Apr 5;18(4):e0281585. doi: 10.1371/journal.pone.0281585 (PMC10075434; doi:10.1371/journal.pone.0281585)
Supplement: S3 File — (PDF) [file pone.0281585.s003.pdf]

Clinical Study Protocol

Glucocorticoid withdrawal and glucocorticoid-induced adrenal insufficiency: a randomized controlled multicenter trial.

Taper Or Abrupt Steroid STop: TOASST trial

Study Type:	Clinical outcome trial with Prednisone or Placebo.
Study Categorisation:	B
Study Registration:	ClinicalTrials.gov Identifier: NCT03153527<br>EUDRA-CT: 2020-005601-48
Study Identifier:	TOASST
Sponsor-Investigator:	Jonas Rutishauser, M.D.<br>Kantonsspital Baden AG, Departement Medizin, Medizinische Klinik<br>CH-5404 Baden, phone: +41-56-486 25 16<br>j.rutishauser@unibas.ch
Investigational Product:	Prednisone
Protocol Version and Date:	Version 3.1, August 24th 2021 (Version for Germany)

Signature Page(s)

Study number	ClinicalTrials.gov Identifier: NCT03153527
	EUDRA-CT: 2020-005601-48
Study Title	Glucocorticoid withdrawal and glucocorticoid-induced adrenal insufficiency: a randomized controlled multicenter trial.

The Sponsor-Investigator and trial statistician have approved the protocol version 3.1 (dated 24.08.2021), and confirm hereby to conduct the study according to the protocol, current version of the World Medical Association Declaration of Helsinki, ICH-GCP guidelines or ISO 14155 norm if applicable and the local legally applicable requirements.

Sponsor-Investigator:
Jonas Rutishauser

Baden,
Place/Date

Signature

Trial statistician:
Marco Cattaneo

Basel,
Place/Date

Signature

Table of Contents

STUDY SYNOPSIS **8**
STUDY SUMMARY IN LOCAL LANGUAGE **10**
ABBREVIATIONS **10**
STUDY SCHEDULE **12**
1. STUDY ADMINISTRATIVE STRUCTURE **13**
1.1 Sponsor-Investigator 13
1.2 Local Principal Investigators 13
1.3 Statistician ("Biostatistician") 14
1.4 Laboratory 14
1.5 Monitoring institutions 14
1.6 Data and Safety Monitoring Board (DSMB) 14
1.7 Any other relevant Committee, Person, Organisation, Institution 15
2. ETHICAL AND REGULATORY ASPECTS **15**
2.1 Study registration 15
2.2 Categorisation of study 15
2.3 Competent Ethics Committee (CEC) 15
2.4 Competent Authorities (CA) 15
2.5 Ethical Conduct of the Study 15
2.6 Declaration of interest 15
2.7 Patient Information and Informed Consent 15
2.8 Participant privacy and confidentiality 16
2.9 Early termination of the study 16
2.10 Protocol amendments 16
3. BACKGROUND AND RATIONALE **17**
3.1 Background and Rationale 17
3.2 Investigational Product (treatment, device) and Indication 17
3.3 Preclinical Evidence 17
3.4 Clinical Evidence to Date 17
3.5 Dose Rationale / Medical Device: Rationale for the intended purpose in study (pre-market MD) 18
3.6 Explanation for choice of comparator (or placebo) 18
3.7 Risks / Benefits 18
3.8 Justification of choice of study population 18
4. STUDY OBJECTIVES **19**
4.1 Overall Objective 19
4.2 Primary Objective 19
4.3 Secondary Objectives 19
4.4 Safety Objectives 19
5. STUDY OUTCOMES **20**
5.1 Primary Outcome 20
5.2 Secondary Outcomes 20
5.3 Other Outcomes of Interest 20
5.4 Safety Outcomes 20

6. STUDY DESIGN 21
6.1 General study design and justification of design 21
6.2 Methods of minimising bias 22
6.2.1 Randomization 22
6.2.2 Blinding procedures 23
6.2.3 Other methods of minimizing bias 23
6.3 Unblinding Procedures (Code break) 23
7. STUDY POPULATION 24
7.1 Eligibility criteria 24
7.2 Recruitment and screening 24
7.3 Assignment to study groups 24
7.4 Criteria for withdrawal / discontinuation of participants 24
8. STUDY INTERVENTION 25
8.1 Identity of Investigational Products (treatment / medical device) 25
8.1.1 Experimental Intervention (treatment) 25
8.1.2 Control Intervention (standard/routine/comparator treatment) 25
8.1.3 Packaging, Labelling and Supply (re-supply) 25
8.1.4 Storage Conditions 25
8.2 Administration of experimental and control interventions 25
8.2.1 Experimental Intervention 25
8.2.2 Control Intervention 25
8.3 Dose modifications 26
8.4 Compliance with study intervention 26
8.5 Data Collection and Follow-up for withdrawn participants 26
8.6 Trial specific preventive measures 26
8.7 Concomitant Interventions (treatments) 26
8.8 Study Drug Accountability 26
8.9 Return or Destruction of Study Drug 27
9. STUDY ASSESSMENTS 28
9.1 Study flow chart(s) / table of study procedures and assessments 28
9.2 Assessments of outcomes 28
9.2.1 Assessment of primary outcome 28
9.2.2 Assessment of secondary outcomes 29
9.2.3 Assessment of other outcomes of interest 29
9.2.4 Assessment of safety outcomes 29
9.2.5 Assessments in participants who prematurely stop the study 30
9.3 Procedures at each visit 30
9.3.1 Screening visit (visit 1) 30
9.3.2 Day 1 (visit 2) 30
9.3.3 Day 7 ± 1d (visit 3) 30
10. SAFETY 31
10.1 Drug studies 31
10.1.1 Definition and assessment of serious adverse events and other safety related events 31
10.1.2 Reporting of Serious Adverse Events (SAE) and other safety related events 31

10.1.3 Follow up of Serious Adverse Events ... 32
11. STATISTICAL METHODS ... 33
11.1 Hypothesis ... 33
11.2 Determination of Sample Size ... 33
11.3 Statistical criteria of termination of trial ... 34
11.4 Planned Analyses ... 34
11.4.1 Datasets to be analysed, analysis populations ... 34
11.4.2 Primary Analysis ... 34
11.4.3 Secondary Analyses ... 34
11.4.4 Interim analyses ... 35
11.4.5 Safety analysis ... 35
11.4.6 Deviation(s) from the original statistical plan ... 35
11.5 Handling of missing data and drop-outs ... 35
12. QUALITY ASSURANCE AND CONTROL ... 36
12.1 Data handling and record keeping / archiving ... 36
12.1.1 Case Report Forms ... 36
12.1.2 Specification of source documents ... 36
12.1.3 Record keeping / archiving ... 36
12.2 Data management ... 36
12.2.1 Data Management System ... 36
12.2.2 Data security, access and back-up ... 36
12.2.3 Analysis and archiving ... 36
12.2.4 Electronic and central data validation ... 36
12.3 Monitoring ... 37
12.4 Audits and Inspections ... 37
12.5 Confidentiality, Data Protection ... 37
12.6 Storage of biological material and related health data ... 37
13. PUBLICATION AND DISSEMINATION POLICY ... 37
14. FUNDING AND SUPPORT ... 37
14.1 Funding ... 37
14.2 Other Support ... 37
15. INSURANCE ... 38
16. REFERENCES ... 38
17. APPENDICES ... 38

STUDY SYNOPSIS

**Sponsor / Sponsor-Investigator**	Prof. Dr. med. Jonas Rutishauser
**Study Title:**	Glucocorticoid withdrawal and glucocorticoid-induced adrenal insufficiency: a randomized controlled multicenter trial.
**Short Title / Study ID:**	Taper Or Abrupt Steroid STop: the TOASST trial.
**Protocol Version and Date:**	Version 3.1; Date: 24.08. 2021
**Trial registration:**	ClinicalTrials.gov Identifier: NCT03153527<br>Eudra CT Nr: 2020-005601-48
**Study category and Rationale**	B; clinical trial with humans testing a drug registered in Switzerland and the European Union (Prednisone) versus placebo.
**Clinical Phase:**	IV
**Background and Rationale:**	No data from controlled trials are available on whether and how to taper glucocorticoids. We aim to establish the safety of rapidly terminating prednisone treatment irrespective of biochemical status of the hypothalamic-pituitary-adrenal axis, provided glucocorticoid cover is ensured in situations of stress. Health conditions are inflammatory, autoimmune, and others except s/p organ transplatation or primary adrenal failure.
**Objective(s):**	1°: To test the hypothesis that in patients treated with systemic glucocorticoids for various inflammatory disorders, rapid termination of treatment will not result in a worse clinical outcome than a tapering regime over four weeks.<br>2°: To test whether the 250 mcg ACTH test predicts the need of unplanned glucocorticoid treatment during 6 months of follow-up. To establish the relationship between clinical signs and symptoms of hypocortisolism and biochemical adrenocortical performance.
**Outcome(s):**	1°: Time to first occurrence of hospitalization, death, initiation of unplanned systemic glucocorticoid therapy, or adrenal crisis.<br><br>Main 2°: Time to first occurrence of individual components of the primary outcome; Cumulative overall systemic glucocorticoid dose; Cumulative systemic glucocorticoid dose administered to treat or prevent adrenal failure; Cumulative systemic glucocorticoid dose administered to treat relapse of disease, specified for each disease
**Study design:**	Randomized controlled noninferiority trial.
**Inclusion / Exclusion criteria:**	Patients with a variety of inflammatory disorders will be eligible, e.g. sarcoidosis, inflammatory bowel disease, polymyalgia rheumatica, rheumatoid arthritis, giant cell arthritis, autoimmune hemolytic anemia, and other autoimmune disease.<br><br>Inclusion criteria: Age ≥ 18 years; daily glucocorticoid dose ≥ 7.5 mg prednisone-equivalent at the time of inclusion; therapy over ≥ 28 days with a cumulative glucocorticoid dose ≥ 420 mg prednisone-equivalent prior to inclusion; tapering not or no longer mandatory to treat underlying disease.<br><br>Exclusion criteria: Primary adrenal failure; pregnancy; treatment with systemic depot glucocorticoids (e.g. intramuscular, epidural); incapability to administer glucocorticoid cover treatment in situations of stress; inability or unwillingness to provide informed consent; untreated hereditary galactose intolerance or lactase deficiency or glucose-galactose malabsorption.

**Measurements and procedures:**	Patients on glucocorticoid treatment (cumulative dose ≥ 420 mg prednisone-equivalent over ≥ 28 days; current daily dose ≥7.5 mg prednisone-equivalent) are eligible if the treating physician deems it feasible to end the glucocorticoid therapy. Participants will be randomized 1:1 (day 0) to abrupt treatment stop (intervention arm) or tapering over 4 weeks (standard treatment arm). A 250 mcg ACTH test will be performed upon randomization, the result of which will be blinded to treating physicians and investigators. Patients will be followed-up by telephone interview (days 7, 35, 90 180). In a subgroup of 180 patients, clinical visits will be performed on days 7 and 35, including an interview, measurement of vital parameters, and a 250 mgc ACTH test.
**Study Product / Intervention:**	Placebo group (intervention arm):<br>Stop glucocorticoid treatment; administer placebo matching the verum preparation in weekly intervals (cf. next section).
**Control Intervention (if applicable):**	Verum group (control/standard arm):<br>If patient is on > 7.5 mg prednisone-equivalent daily: administer 7.5 mg q.d. for 7 days, then 5 mg q.d. for 7 days, then 2.5 mg q.d. for 7 days, then 2.5 mg q.d.every other day for 7 days, then stop. If patient on 7.5 mg q.d.: maintain for 7 days, then taper as above.
**Number of Participants with Rationale:**	Total number of participants in both arms: 573 (95% CI: 565 to 580).
**Study Duration:**	7.5 years
**Study Schedule:**	First-Participant-In: June 2017<br>Last-Participant-Out (planned): December 2024
**Local Pricipal Investigators:**	Prof. Philip Schütz, Aarau; Prof. Marc Donath, Basel; Prof. Jörg Leuppi, Liestal; Prof. Michael Brändle, St. Gallen; Prof. F. Beuschlein, Zürich; Prof. Andreas Zeller, Liestal; Prof. Daniel Aeberli, Bern; Prof. Gottfried Rudofsky, Olten; Prof. J.-L. Reny, Genève, PD A. Kistler, Frauenfeld, Prof. Robert Thurnheer, Münsterlingen; PD Dr. Stephan Böhm, Bülach, Dr. med. Irina Chifu (Würzburg), Prof. Nicole Reisch, München; Prof. Jörg Bojunga, Frankfurt, Prof. Jonas Rutishauser, Baden (Sponsor/Investigator), further<br><br>For full contact details please see p. 14/15.
**Study Centers:**	Department of Internal Medicine, Kantonsspital Baden AG, 5404 Baden<br>Department of Internal Medicine, Kantonsspital Frauenfeld, 8501 Frauenfeld<br>Department of Internal Medicine, Kantonsspital Münsterlingen, 8596 Münsterlingen<br>Department of Internal Medicine, Tellstrasse, 5001, Kantonsspital, Aarau<br>Clinic for Endocrinology/Diabetology/Metabolism, University Hospital, 4031 Basel<br>Department of Medicine, Kantonsspital Baselland, Site 4410 Liestal<br>Department of Internal Medicine, Kantonsspital, 9007 St. Gallen<br>University Center for Primary Health Care (uniham-bb), Kantonsspital Baselland, 4410 Liestal<br>Department of Rheumatology, Immunology, and Allergology, Inselspital, 3010 Bern<br>Department of Medicine, Metabolic Unit, Kantonsspital, 4600 Olten<br>Dept. of Endocrinology, Diabetology and Clinical Nutrition, University Hospital, 8091 Zürich<br>Department of Internal Medicine, University Hospitals Geneva, 1205 Geneva<br>University Hospital Würzburg,97080 Würzburg
**Statistical Considerations:**	A sample size of n=573 was calculated aiming at a power of 80%, defining the significance level at 5%, assuming an event rate of 40% in each arm, and a drop-out rate of 10% over 6 months. Following a modified Delphi procedure among clinical experts, we defined noninferiority as an absolute increase in the event rate by 13% over 6 months. Noninferiority will be concluded if the upper limit of the two-sided 95% confidence interval of the hazard ratio between the experimental and the control arm lies below the critical value. For the above scenario, this value is 1.478.

GCP Statement:	This study will be conducted in compliance with the protocol, the current version of the Declaration of Helsinki, the ICH-GCP or ISO EN 14155 (as far as applicable) as well as all national legal and regulatory requirements.

STUDY SUMMARY IN LOCAL LANGUAGE

Cortisol ist ein körpereigenes, in den Nebennieren gebildetes Hormon, das wichtige Stoffwechselfunktionen steuert und in Stresssituationen zur Erhaltung eines ausreichenden Blutdrucks und Blutzuckerspiegels dient. Prednison und andere synthetisch hergestellte Cortisol-ähnliche Medikamente werden zur Entzündungshemmung bei einer Vielzahl von Krankheiten in Tablettenform oder als Injektionen verabreicht. Beispiele sind rheumatische Erkrankungen, entzündliche Darmerkrankungen, gewisse Erkrankungen des blutbildenden Systems, Erkrankungen des Nervensystems, oder Tumorerkrankungen. Prednison hat viele unerwünschte Arzneimittel-Wirkungen, wie Gewichtszunahme, Schwächung von Knochen- und Bindegewebe, Erhöhung des Blutdrucks, oder erhöhte Blutzuckerspiegel bis hin zum Diabetes. Eine oft beobachtete Nebenwirkung von Prednison ist die Unterdrückung der körpereigenen Produktion von Cortisol. Dieser Effekt dauert über die Beendigung der Prednisontherapie hinaus an. Es ist im Einzelfall bisher jedoch nicht möglich vorherzusagen, ob eine solche Unterdrückung der Nebennierenfunktion auftreten wird oder nicht und wie lange eine solche ggf. dauern wird. Deswegen führt man in der klinischen Medizin vor der geplanten Beendigung der Prednisontherapie oft einen Labortest durch, welcher die Frage beantwortet. Bei diesem sogenannten Synacthentest spritzt man ein zweites Hormon, Synacthen, welches die Nebennieren zur Cortisolproduktion und –ausschüttung in die Blutbahn ankurbelt, und misst den Cortisolspiegel im Blut 30 und 60 Minuten nach der Injektion (Deutschland: 0 und 60 Minuten). Sind die gemessenen Cortisolspiegel erniedrigt, wird Prednison nicht abrupt abgesetzt, sondern die Dosis in kleinen Schritten reduziert, bevor es nach Wochen schliesslich ganz gestoppt wird. Dieses Vorgehen ist zwar medizinisch einleuchtend, wissenschaftlich bislang aber nicht belegt. Es ist nicht untersucht, ab welcher Dosis, in welchen Schritten und über welche Zeitdauer die Prednison-Medikation ausgeschlichen werden soll. Es ist nicht einmal klar, ob ein Ausschleichen überhaupt nötig ist, auch wenn im Synacthentest tiefe Cortisolspiegel gemessen werden. In einer eigenen Untersuchung mit über 200 Patienten, die mit Prednison behandelt wurden, haben wir auch bei ungenügendem Resultat im Synacthentest und trotz abrupter Beendigung der Therapie keinerlei ungünstigen Folgen beobachtet, und die gezielte Befragung nach Symptomen eines Cortisolmangels ergab keinen Unterschied zu Personen mit normalem Testresultat. Es ist also davon auszugehen, dass in vielen Fällen, in denen Prednison ausgeschlichen wird, unnötigerweise eine wochenlange Verlängerung der Behandlung stattfindet, was bekanntermassen die Nebenwirkungsgefahr erhöht.

Die vorliegende Studie soll deshalb die Hypothese prüfen, dass auch nach längerer Behandlungsdauer mit Prednison (mindestens über 4 Wochen mit insgesamt mindestens 420 Milligramm) das Medikament abrupt gestoppt werden darf, ohne dass dadurch ein schlechterer Verlauf resultiert. Die Beobachtungsdauer ist 6 Monate nach Studieneinschluss. Um eine genügende Aussagekraft zu erhalten, werden 573 Patienten für die Untersuchung benötigt, wovon die Prednisonbehandlung bei der einen Hälfte über vier Wochen ausgeschlichen und bei der anderen abrupt gestoppt wird. Teilnehmer der letzteren Gruppe erhalten statt Prednison gleich aussehende Schein-Präparate (Placebo). Über die Gruppenzuteilung entscheidet der Zufall, und weder Teilnehmer noch Prüfarzt kennen die Gruppenzugehörigkeit (Doppelblind-Prinzip). Zur Beurteilung des Verlaufs wird in beiden Gruppen die Zeit verglichen, die verstreicht bis zum erstmaligen Auftreten eines oder mehrerer der folgenden Ereignisse: Hospitalisation, Anzeichen eines Cortisolmangels, Tod, oder ungeplante Wiederbehandlung mit Prednison oder einem anderen Cortisol-ähnlichen Medikament (zum Beispiel wegen eines Rückfalls der ursprünglich mit Prednison behandelten Krankheit). Es soll zudem untersucht werden, ob der Synacthentest geeignet ist, das Auftreten dieser Ereignisse vorherzusagen. An alle Teilnehmenden werden sicherheitshalber Prednisontabletten abgegeben; diese sind in Situationen mit erhöhtem Cortisolbedarf, zum Beispiel bei fieberhaften Erkrankungen, einzunehmen (sog. Stressprophylaxe).

Dies ist die erste Studie überhaupt, in der das Absetzen von Prednison mit einem Ausschleich-Schema direkt verglichen wird. Bestätigt sich die Studienhypothese, kann künftig auf die unnötige Verlängerung von Prednisonbehandlungen verzichtet werden, was angesichts des ungünstigen Nebenwirkungsprofils wünschbar wäre. Falls sich zeigt, dass das abrupte Stoppen doch Nachteile hat gegenüber dem Ausschleichen, gäbe es auch für letzteres Vorgehen erstmals eine wissenschaftlich fundierte Grundlage.

ABBREVIATIONS

AE	Adverse Event
CA	Competent Authority (e.g. Swissmedic,BfArM)
CEC	Competent Ethics Committee
CRF	Case Report Form
ClinO	Ordinance on Clinical Trials in Human Research *(in German: KlinV, in French: OClin)*
eCRF	Electronic Case Report Form

CTCAE	Common terminology criteria for adverse events
DSUR	Development safety update report
GCP	Good Clinical Practice
IB	Investigator’s Brochure
Ho	Null hypothesis
H1	Alternative hypothesis
HFG	Humanforschungsgesetz (Law on human research)
HMG	Heilmittelgesetz
HRA	Federal Act on Research involving Human Beings
IMP	Investigational Medicinal Product
IIT	Investigator-initiated Trial
ISO	International Organisation for Standardisation
ITT	Intention to treat
KlinV	Verordnung über klinische Versuche in der Humanforschung *(in English: ClinO, in French OClin)*
LPTh	Loi sur les produits thérapeutiques
LRH	Loi fédérale relative à la recherche sur l’être humain
MD	Medical Device
OClin	Ordonnance sur les essais cliniques dans le cadre de la recherche sur l'être humain *(in German : KlinV, in English : ClinO)*
PI	Principal Investigator
SDV	Source Data Verification
SOP	Standard Operating Procedure
SPC	Summary of product characteristics
SUSAR	Suspected Unexpected Serious Adverse Reaction
TMF	Trial Master File

STUDY SCHEDULE

Study Periods	Screening	Treatment, Intervention Period			Follow-up		
Visit	1	2	3	4	5	6	7 (final)
Time (hour, day, week)	-7d to -1d	Day 1	Day 7 ±1d	Day 28 ±3d	Day 35 ±3d	Day 90 ± 5d	Day 180 ±7d
Patient Information and Informed Consent	x						
In- /Exclusion Criteria	x						
Vital Signs (heart rate, blood pressure), body weight, height [only day 1])		x	x **		x **		
Laboratory Tests:<br>250 mcg Synacthen® test;		x	x **		x **		
Pregnancy test in menstruating women	x						
Randomisation		x					
Interview Assessment: Primary outcome variables*† - fill out CRF			x	x	x	x	x
Interview Assessment: Secondary outcome variables*†- fill out CRF			x	x	x	x	x
Interview Assessment:*†: symptoms & signs of hypocortisolism; concomitant therapy; serious adverse events; therapeutic interventions by other physicians – fill out CRF		x	x	x	x	x	x
Dispense and start study medication		x					
Terminate study medication				x			
Return study medication††					x††		

* Assessed in all study participants
** Due to restricted resources, assessed only in a subgroup of 180 patients living in the vicinity of their respective study center.
† Assessed by telephone, except day 1 (all centers) and days 7 and 35 (subgroup of 180 patients that have 2 clinical visits during follow-up).
†† by mail, except if a clinical visit is performed on day 35.

1. STUDY ADMINISTRATIVE STRUCTURE

Planning Committee:
Responsible: Jonas Rutishauser, Kantonsspital Baden AG, Medizinische Klinik, CH-5404 Baden, Phone +41-56-486 25 16, Fax +41-56-486 21 28, j.rutishauser@unibas.ch
Clinical Expert: Philip Schütz, Kantonsspital Aarau, Medizinische Universitätsklinik, Tellstrasse, 5001 Aarau, Phone +41 62 838 95 24, Fax +41 62 838 69 45, schuetzph@gmail.com
Clinical Expert: Christoph Henzen, Departement Medizin, Luzerner Kantonsspital, 6000 Luzern 16, Phone +41-41-205 51 03, Fax +41-41-205 51 84, christoph.henzen@luks.ch
Clinical Expert: Michael Brändle, Departement Medizin, Kantonsspital, 9007 St. Gallen, Phone +41-71-494 11 11, Fax +41-71 494 61 21, michael.braendle@kssg.ch

Data and safety monitoring board (DSMB):
Prof. Christoph Beglinger, M.D., Bruggweg 28, 4144 Arlesheim, Phone +41-61-701 20 60, christoph.beglinger@unibas.ch
Prof. Jürgen Drewe, Dr. pharm., Department of Clinical Pharmacology, University Hospital, Petersgraben 4, 4031 Basel, Phone +41- 78 923 27 44, juergen.drewe@unibas.ch,
Prof. Jörg Goldhahn, M.D., ETH Zürich D-HEST, HCP H15.3, Leopold Ruzicka-Weg 4, 8093 Zürich, Phone +41-44-633 20 69, jgoldhahn@eth.ch
Sabine Schaedelin, MSc, Statistician, Clinical Trial Unit (CTU), University Hospital Basel, Spitalstrasse 12, 4031 Basel, Phone: +41 61 556 51 67, sabine.schaedelin@usb.ch

1.1 Sponsor-Investigator

Jonas Rutishauser, Kantonsspital Baden AG, Medizinische Klinik, CH-5404 Baden, Phone +41-56-486 25 16, Fax +41-56-486 21 28, j.rutishauser@unibas.ch
The sponsor investigator was responsible for the trial design and will participate in patient recruitment, data collection, analysis and interpretation of data. He will be the responsible author in the final report. He will also be responsible for the communication with the CTU of the University Hospital Basel, who will be responsible for the data management.

1.2 Local Principal Investigators

Trial Site Kantonsspital Baden:
Jonas Rutishauser, Kantonsspital Baden AG, Medizinische Klinik, CH-5404 Baden, Phone +41-56-486 25 16, Fax +41-56-486 21 28, j.rutishauser@unibas.ch

Trial Site Kantonsspital Aarau:
Philipp Schütz, Kantonsspital Aarau, Department of Internal Medicine, Tellstrasse, 5001 Aarau, Phone +41 62 838 95 24, Fax +41-62-838 69 45, schuetzph@gmail.com

Trial Site University Hospital Basel:
Marc Donath, Endocrinology/Diabetology/Metabolism, Petersgraben 4, 4031 Basel, Phone +41 61 265 50 78, Fax +41 61 265 51 00, marc.donath@usb.ch

Trial Site Kantonsspital Baselland, Liestal:
Jörg Leuppi, Internal Medicine, Kantonsspital Baselland/Liestal; 4410 Liestal; Phone +41-61-925 21 80, Fax: +41-61-925 28 04, joerg.leuppi@ksbl.ch

Trial Site Kantonsspital St. Gallen:
Michael Brändle, Department of Internal Medicine, Kantonsspital, 9007 St. Gallen, Phone +41-71-494 31 16, Fax +41-71-494 61 21, michael.braendle@kssg.ch

Trial Site Institute for Primary Health Care, Basel University:
Andreas Zeller, Universitäres Zentrum für Hausarztmedizin beider Basel | uniham-bb, Kantonsspital Baselland, Rheinstr. 26, 4410 Liestal, phone: +41-61-925 20 75, Fax: +41- 61 925 28 68, andreas.zeller@unibas.ch

Trial Site University Hospital Bern, Inselspital:
Daniel Aeberli, Department of Rheumatology, Immunology, and Allergology, Inselspital, Haus 5, Eingang 16p, Stock F, F14, 3010 Bern, Phone: +41-31- 632 07 32, daniel.aeberli@insel.ch

Trial Site Kantonsspital Frauenfeld:
Andreas Kistler, Department of Internal Medicine, Pfaffenholzstrasse 4, 8501 Frauenfeld
Phone: +41 52 723 76 43; andreas.kistler@stgag.ch

Trial Site Kantonsspital Münsterlingen:
Robert Thurnheer, Departement of Internal Medicine, Spitalcampus 1, 8596 Münsterlingen
Phone: +41 71 686 21 75; robert.thurnheer@stgag.ch

Trial Site University Hospital Zürich:
Felix Beuschlein, Department of Endocrinology, Diabetology and Clinical Nutrition, Universitätsspital Zürich, 8091 Zürich, Phone: +41-44-255 36 25; Fax: +41-44-255 44 47; felix.beuschlein@usz.ch

Trial Site Kantonsspital Olten:
Gottfried Rudofsky, Stoffwechselzentrum, Kantonsspital Olten, 4600 Olten; Phone: +41-62-311 44 94, gottfried.rudofsky@spital.so.ch

Trial Site University Hospital Geneva;
Jean-Luc Reny, Division of Internal Medicine, Rue Gabrielle Perret-Gentil 4, 1205 Geneva
Phone: +41 22 372 90 52; Fax: +41 22 372 91 16

Trial Site Spital Bülach AG
Stephan Böhm, Division of Gastroenterology, Spitalstrasse 24, 8180 Bülach
Phone: +41 44 863 2966; Fax: + 41 (0) 44 863 2967; stephan.boehm@spitalbuelach.ch

Trial Site University Hospital Würzburg
Irina-Oana Chifu, MD, Dept. of Medicine I, Oberduerrbacher Str. 6
97080 Wuerzburg, Germany
Phone. +49-931-201-39201; Fax +49-931-201-6039021; email: Chifu_I@ukw.de

Trial Site München
Nicole Reisch, MD, Medizinische Klinik und Poliklinik IV, Endokrinologische Forschung I Endokrinologische Ambulanz
Campus Innenstadt, Ziemssenstr. 1 80336 München, Germany
Phone +49- 89 4400-52237; Fax +49- 9 4400-52408; email: Nicole.Reisch@med.uni-muenchen.de

Trial Site Frankfurt
Jörg Bojunga, MD, Universitätsklinikum Frankfurt, Zentrum für Innere Medizin, Medizinische Klinik I, Schwerpunkt Endokrinologie, Theodor-Stern-Kai 7, 60590 Frankfurt, Germany
Phone +49- 69 6301-5396; Fax +49- 69 6301-6405; email: Joerg.Bojunga@kgu.de

1.3 Statistician ("Biostatistician")

Marco Cattaneo, PhD, University Hospital Basel, Departement Klinische Forschung, Clinical Trial Unit (CTU) Spitalstrasse 12, CH-4031 Basel, Phone: +41 (0)61 556 54 98, Fax: +41 (0)61 265 94 10, marco.cattaneo@usb.ch

1.4 Laboratory

Core Lab for ACTH test / cortisol measurements; Viollier AG, Hagmattstrasse 14, CH-4123 Allschwil, Phone +41-61-486 11 11

1.5 Monitoring institutions

University Hospital Basel, Clinical Trial Unit, Schanzenstrasse 55, 4031 Basel.
Study coordinator Department of Medicine, Kantonsspital Baselland, Rheinstrasse 26, 4410 Liestal

1.6 Data and Safety Monitoring Board (DSMB)

Prof. Christoph Beglinger, M.D., St. Claraspital, Kleinriehenstrasse 30, Postfach, 4058 Basel, Phone +41-61-685 86 31, Fax +41-61-691 95 18, christoph.beglinger@unibas.ch

Prof. Jürgen Drewe, Ph.D., Department of Clinical Pharmacology, University Hospital Basel, Petersgraben 4, 4031 Basel, Phone +41 78 923 27 44, juergen.drewe@unibas.ch

Prof. Jörg Goldhahn, M.D., ETH Zürich D-HEST, HCP H15.3, Leopold Ruzicka-Weg 4, 8093 Zürich, Phone +41-44-633 20 69, jgoldhahn@eth.ch

Sabine Schaedelin, MSc., Statistician CTU Basel, University Hospital Basel, Spitalstrasse 12, 4031 Basel Phone +41 61 556 51 67, Fax +41 61 556 54 98, sabine.schaedelin@usb.ch

The Data and Safety Monitoring Board will perform the first examination of data 6 months after 100 patients have been included. At this time, the event rate is estimated to be 40. After this, yearly meetings will be performed. An SOP will be established regarding the organization of the DSMB and the reporting structure. The DSMB is independent of the sponsor/investigator, its members are not involved in the conduct of the study and have no competing interests.

1.7 Any other relevant Committee, Person, Organisation, Institution

N.a.

2. ETHICAL AND REGULATORY ASPECTS

Before the study will be conducted, the protocol, the proposed patient information and consent form as well as other study-specific documents shall be submitted to the EKNZ (Ethikkommission Nordwest- und Zentralschweiz) and to the Swiss Agency for Therapeutic Products (Swissmedic).

2.1 Study registration

The study is registered with clinicaltrials.gov (identifier: NCT03153527) and Eudra CT Number: 2020-005601-48. **In addition, the study is registered in a national language in the Swiss Federal Complementary Database (Portal) of the Federal Office of Public Health (www.kofam.ch) SNCTP000002172**

2.2 Categorisation of study

This is a clinical trial of category B as assessed with the KOFAM online form (Termination of treatment with a pharmacological agent registered in Switzerland is evaluated in humans; the drug is prescribed for registered indications by treating physicians outside the study team; the trial includes a placebo control).

2.3 Competent Ethics Committee (CEC)

The responsible investigator at each site ensures that approval from an appropriately constituted Competent Ethics Committee (CEC) is sought for the clinical study.

No changes are made to the protocol without prior Sponsor and CEC approval, except where necessary to eliminate apparent immediate hazards to study participants.

Premature study end or interruption of the study is reported within 15 days. The regular end of the study is reported to the CEC within 90 days, the final study report shall be submitted within one year after study end. Amendments are reported according to chapter 2.10.

2.4 Competent Authorities (CA)

The Sponsor will obtain approval from all Competent Authority (CA) of the concerned Member States before the start of the clinical trial. Clinical trial authorisation application approval must be received from all Regulatory Agencies before the start of the study.

Any changes in the research activity and all unanticipated problems involving risks to humans will be reported to the CA. No substantial amendments to the protocol will be made without prior approval by the CA, except where necessary to eliminate apparent immediate hazards to Study Participants. Non-substantial protocol amendments will be reported to the CEC according to section **Error! Reference source not found.**

Deleted

Premature study end or interruption of the study is reported within 15 days. The regular end of the study is reported to the CA within 90 days, the final study report shall be submitted within one year after study end. Amendments are reported according to chapter 2.10; non-substantial amendments shall be reported as soon as possible.

2.5 Ethical Conduct of the Study

The study will be carried out in accordance to the protocol and with principles enunciated in the current version of the Declaration of Helsinki, the guidelines of Good Clinical Practice (GCP) issued by ICH, in case of medical device: the European Directive on medical devices 93/42/EEC and the ISO Norm 14155 and ISO 14971, the Swiss Law and Swiss regulatory authority's requirements. The CEC and regulatory authorities will receive annual safety and interim reports and be informed about study stop/end in agreement with local requirements.

2.6 Declaration of interest

There is no conflict of interest.

2.7 Patient Information and Informed Consent

The investigators will explain to each participant the nature of the study, its purpose, the procedures involved, the expected duration, the potential risks and benefits and any discomfort it may entail. Each participant will be

informed that the participation in the study is voluntary and that he/she may withdraw from the study at any time and that withdrawal of consent will not affect his/her subsequent medical assistance and treatment.
The participant must be informed that his/her medical records may be examined by authorised individuals other than their treating physician.
All participants for the study will be provided a participant information sheet and a consent form describing the study and providing sufficient information for participant to make an informed decision about their participation in the study. Enough time will be given to the participant to decide whether to participate or not, "enough" meaning up to several days since inclusion is not tied to a situation of medical emergency.
The patient information sheet and the consent form will be submitted to the CEC and to the competent authority to be reviewed and approved. The formal consent of a participant, using the approved consent form, must be obtained before the participant is submitted to any study procedure.
The participant should read and consider the statement before signing and dating the informed consent form, and should be given a copy of the signed document. The consent form must also be signed and dated by the investigator (or his designee) and it will be retained as part of the study records.

2.8 Participant privacy and confidentiality

The investigator affirms and upholds the principle of the participants' right to privacy and shall comply with applicable privacy laws. Especially, anonymity of the participants shall be guaranteed when presenting the data at scientific meetings or publishing them in scientific journals.
Individual subject medical information obtained as a result of this study is considered confidential and disclosure to third parties is prohibited. Subject confidentiality will be further ensured by utilising subject identification code numbers to correspond to treatment data in the computer files.
For data verification purposes, authorised representatives of the Sponsor-Investigator, a competent authority (e.g. Swissmedic), or an ethics committee may require direct access to parts of the medical records relevant to the study, including participants' medical history.

2.9 Early termination of the study

The Sponsor-Investigator and any competent authority may terminate the study prematurely according to certain circumstances, for example:

- ethical concerns,
- insufficient participant recruitment,
- when the safety of the participants is doubtful or at risk, respectively,
- alterations in accepted clinical practice that make the continuation of a clinical trial unwise,
- early evidence of benefit or harm of the experimental intervention

2.10 Protocol amendments

Substantial amendments are only implemented after approval of the CEC and CA respectively.
Under emergency circumstances, deviations from the protocol to protect the rights, safety and well-being of human subjects may proceed without prior approval of the sponsor and the CEC/CA. Such deviations shall be documented and reported to the sponsor and the CEC/CA as soon as possible.
All Non-substantial amendments are communicated to the CA as soon as possible if applicable and to the CEC within the Annual Safety Report (ASR).

3. BACKGROUND AND RATIONALE

3.1 Background and Rationale

To date, there are no published RCTs comparing glucocorticoid tapering with rapid stopping, with regard to hazards resulting from adrenal failure or to disease relapse. Data from trials comparing different tapering schemes in patients treated for inflammatory or autoimmune disorders are scarce and inconclusive. A systematic review found “insufficient evidence about the efficacy and safety of different glucocorticoid withdrawal regimens” and demonstrated “a research gap of high quality randomized trials in a wide range of chronic disorders.”[1] The review evaluated 9 trials (Crohn’s disease: 1; graft versus host disease: 1; bronchial asthma: 5; COPD: 2), 7 of which included less than 50 patients. We have performed an actual literature search in PubMed and have found no systematic reviews or meta-analyses published after the report by Richter et al. Given the very small scientific evidence base for the common clinical situation of glucocorticoid withdrawal and the potential for serious untoward effects of these drugs, a RCT should be conducted. From previous own data, we have no evidence that abrupt withdrawal will endanger patients with acute exacerbation of COPD, even in the presence of biochemical adrenal insufficiency.[2,3] This is in line with data from literature from the 1960’s and 1970s. In one study with 21 rheumatoid arthritis patients under chronic glucocorticoid treatment, therapy was stopped abruptly 18-48 hours prior to synovectomy of the knee without intraoperative glucocorticoid cover.[4] Only one patient developed glucocorticoid-sensitive hypotension during the operation. In another study with 48 glucocorticoid-treated patients (duration, ½ month to >20 years) undergoing major surgery, the stimulated plasma corticosteroid concentration in a preoperative 250 mcg corticotropin test correlated highly significantly with that measured intraoperatively. Patients were boldly taken off glucocorticoid treatment 36 hours prior to surgery. However, none of 31 patients with a blunted preoperative response to corticotropin showed manifest intra- and perioperative adrenal insufficiency or needed cortisol substitution, despite a high proportion (80%) of insufficient corticosteroid levels 1 hour after incision.[5] Thus, the relevance of both the ACTH stimulation test and any of the numerous tapering regimens used in clinical routine are unclear. However, it is important to note that potential hazards of adrenal insufficiency will not be underestimated in the current trial. Treating physicians will be informed about their patients’ participation in the trial, and patients will be instructed on when and how to use rescue glucocorticoids. These safety measures were also taken in our previous “REDUCE” trial.[2]

From our own observations on adrenal function in participants of the “REDUCE” trial[2] and previously published literature, we have generated the hypothesis that in patients treated with systemic glucocorticoids for a variety of disorders, it is feasible and safe to rapidly terminate steroid therapy irrespective of biochemical status of the HPA axis, provided glucocorticoid cover is ensured in situations of stress. The primary purpose of the study is to establish feasibility and safety of rapidly terminating systemic glucocorticoid treatment, irrespective of biochemical status of the HPA axis. The secondary purpose of the study is to assess the usefulness of the 250 mcg ACTH test to predict the clinical outcome with respect to the primary outcome.

3.2 Investigational Product (treatment, device) and Indication

Standard treatment arm: Prednisone, a synthetic glucocorticoid with intermediate duration of action (biological half-life 18-35 hrs). The detailed characteristics of the compound, marketed under several brand names, can be found at www.swissmedicinfo.ch: Prednison Streuli (Swissmedic registration number: 29349); Prednison Axapharm (Swissmedic registration number: 58761); Prednison Galepharm (Swissmedic registration number: 50821). In Germany, the compound is also marketed under several brand namens, e.g. Prednison Galen (registration number for 5 mg strength: 33644.00.00), Prednison Decortin (registration number for 5 mg strength: 8452.00.00), or Prednison Acis (registration number for 5 mg strength: 49572.00.00).
)

Interventional treatment arm: matching placebo.

3.3 Preclinical Evidence

Not applicable.

3.4 Clinical Evidence to Date

The clinical research data pertaining to the current protocol is discussed under chapter 3.1.

3.5 Dose Rationale / Medical Device: Rationale for the intended purpose in study (pre-market MD)

At the time of inclusion, patients must be on a dose low enough to permit an abrupt stop, but high enough to still suppress the HPA axis. Based on these considerations, the planning committee settled on an a daily dose of ≥7.5 mg prednisone-equivalent at the time of inclusion and on a total treatment duration of ≥ 28 days, with a minimal cumulative dose of 420 mg, as prerequisite for eligibility.

3.6 Explanation for choice of comparator (or placebo)

Although ideally chosen for the standard arm in a noninferiority trial, there is no published standard, “best-of-care” glucocorticoid tapering scheme, but a large variety of expert opinions. Accordingly, for many inflammatory and autoimmune disorders, there are no specific guidelines as to the tapering regimens. Also, there are no controlled data indicating the superiority, with respect to clinical outcome of the underlying disease, of one particular tapering scheme over another, or of tapering versus abrupt stopping. These facts and our own observations in COPD patients with biochemically suppressed adrenal glands have led us to hypothesize that, in patients chronically treated with glucocorticoids, the clinical outcome will not be worse if glucocortidoids are stopped abruptly than if they are tapered over as long as 4 weeks. This hypothesis can only be tested in a randomized, placebo-controlled trial. Hence we chose placebo as comparator. This is ethically justifiable because the dangers of clinically relevant secondary adrenal failure are small (see chapter 3.1. above). Should signs and/or symptoms of glucocorticoid deficiency nevertheless occur, or should the patient encounter circumstances with increased needs for glucocortisoids (e.g. an acute illness) or undergo a stressful procedure (e.g. an operation), glucocortoicoid cover is given as per protocol.

3.7 Risks / Benefits

Potential risks:

In the standard treatment (prednisone) arm, the potential risks are those of unwarranted and prolonged glucocorticoid therapy, in particular: weight gain, osteopenia/osteoporosis, hyperglycemia, hypertension, muscle catabolism, increased susceptibility to infections due to immunosuppression, and suppression of the endogenous cortisol production by the adrenals.

In the intervention (placebo) arm, there is a potential risk of flare-up of the inflammatory disorder (although this is not based by high-grade evidence), and of unforeseen glucocorticoid requirements, be it due to flare-up of the underling disease, glucocorticoid deficiency, or glucocorticoid withdrawal syndrome. The risk of endangering the patients is offset by the ease of glucocorticoid stress prophylaxis and cover in case of signs and symptoms of hypocortisolism, for which patients will be instructed and provided with en emergency dose of five 10 mg tablets of prednisone.

Trial participants in both treatment arms may be at risk for severe courses of coronavirus disease-19 (COVID-19), be it due to immunosuppression following longstanding glucocorticoid treatment prior to study enrolment, or due to iatrogenic hypocortisolism, or both. While COVID-19 vaccination is not mandatory for trial participation, patients should be encouraged to undergo vaccination. All participants are carefully instructed regarding emergency glucocorticoid therapy. This so-called stress prophylaxis should also be taken by the patient should post-vaccination symptoms of viral disease occur, but not routinely in order not to jeopardize efficacy of the vaccine. The “TOASST” trial will be conducted in accordance with all current instructions by the competent authorities.

Potential benefits:

The main overall benefit of this trial is the generation of high-level evidence about the feasibility and safety of rapid glucocorticoid withdrawal in comparison with a tapering regime, as no published data on this topic exists from randomized controlled trials.

In the intervention (placebo) arm, there is the potential benefit of reducing the cumulative prednisone dose, thereby minimizing the exposure to as well as untoward effects of glucocorticoids. Other benefits include the generation of evidence regarding the potential of the 250 mcg ACTH test to stratify patients regarding the need for tapering and the prediction of future glucocorticoid demand; as well as regarding the correlation of biochemical and clinical parameters of adrenal insufficiency.

We know of no competing trial to our study.

3.8 Justification of choice of study population

Patients will be recruited outside emergency situations, and no vulnerable participants will be recruited.

The eligible study population consists adult (≥18 year-old) patients who are under glucocorticoid treatment due to an autoimmune or inflammatory disease for which tapering schemes are not standardized, such as rheumatoid arthritis, polymyalgia rheumatica, giant cell arteritis, brain edema due to tumor metastases, sarcoidosis, asthma, autoimmune hemolytic anemia, inflammatory bowel disease, and other disorders. The treating physician will decide whether the acitivity of the underlying disorder permits a potential abrupt treatment stop. At the time of inclusion, patients must be on an oral dose low enough to warrant the abrupt stop, but high enough to still suppress the HPA axis, i.e. corresponding to ≥. mg prednisone-equivalent daily. Glucocorticoid therapy must have lasted for at least 28 days prior to inclusion and amounted to a cumulative dose of ≥420 mg prednisone-equivalent, The average daily dose must be ≥7.5 mg prednisone-equivalent.

Patients are not eligible to participate in the trial if they are treated with glucocorticoids for primary adrenal failure or to prevent solid or transplant graft rejection, if they are pregnant, if they are incapable or unwilling to administer glucocorticoid cover treatment in situations of stress, or if the systemic depot glucocorticoids were administered by an non-oral route (e.g. intramuscular, epidural).

4. STUDY OBJECTIVES

4.1 Overall Objective

To perform the first adequately powered randomized, placebo-controlled, multicenter noninferiority trial, comparing rapid termination of systemic glucocorticoid treatment with a tapering regime over 4 weeks.

4.2 Primary Objective

The primary objective of this trial is to establish the feasibility and safety of rapidly terminating systemic glucocorticoid treatment, irrespective of biochemical status of the HPA axis.

4.3 Secondary Objectives

The secondary objectives of this trial are:

- To establish the clinical relevance of the 250 mcg ACTH (Synacthen®) stimulation test.
- To establish the relationship between clinical signs and symptoms of hypocortisolism and biochemical adrenocortical performance

4.4 Safety Objectives

The study aims to assess the safety of rapidly terminating chronic glucocorticoid therapy in a setting where patients are instructed about the signs and symptoms of hypocortisolism and provided with an emergency dose of five 10 mg tablets of prednisone to cover increased glucocorticoid needs.

5. STUDY OUTCOMES

5.1 Primary Outcome

The composite primary outcome is:
Time to first occurrence of hospitalization, death, initiation of unplanned systemic glucocorticoid therapy, or adrenal crisis (defined as glucocorticoid-responsive hypotension or shock with or without accompanying symptoms and signs such as weakness, apathy, nausea, vomiting, abdominal pain, hypothermia, hyponatremia [serum sodium < 135 mM], hyperkalemia [serum potassium > 5 mM], hypoglycemia [plasma glucose < 3.5 mM]); whichever occurs first.

5.2 Secondary Outcomes

1. Time to first occurrence of individual components of the primary outcome;

2. Cumulative overall systemic glucocorticoid dose;

3. Cumulative systemic glucocorticoid dose administered to treat or prevent adrenal failure;

4. Cumulative systemic glucocorticoid dose administered to treat relapse of disease, specified for each disease;

5. General health status as self-assessed by the participant on a visual analog scale (VAS) from 0 to 100, assessed at days 1, 7, 28, 35, 90,180;

6. Score of symptoms and signs of hypocortisolism: weakness, hypothermia, nausea, vomiting, abdominal pain, fatigue, dizziness (assessed in structured interviews at time points: days 1, 7, 28, 35, 90,180). Blood pressure (supine and standing) is assessed day 1 in all participants, but due to limited resources only in a subgroup of 180 patients living near their respective study center on days 7and 35.

7. Performance in 250 mcg ACTH (Synacthen®) test: test assessed day 1 in all participants, plus days 7 and 35 in a subgroup of 180 patients,

8. In patients hospitalized at study entry: length of hospital stay.

5.3 Other Outcomes of Interest

n.a.

5.4 Safety Outcomes

The individual components of the primary outcome are defined as safety outcomes. The occurrence of an adrenal crisis is of particular relevance due to its obvious danger for the patient. At the same time, papers reporting unequivocal, non-circumstancial evidence of true glucocorticoid-responsive shock in **secondary** (glucocorticoid-induced) adrenal insufficiency are exceedingly rare[8] and date back well into the last century.[9,4]. Because it is not realistic to choose this event as single endpoint, it was defined as one component of the composite primary outcome.
Judging from the literature, it is likely that the imminent danger of adrenal crisis in secondary adrenal insufficiency is overestimated quantitatively. This is supported by our own data from the REDUCE trial.[3] As in the REDUCE trial, patients in this trial will receive a written instruction on signs and symptoms of hypocortisolism and will be provided with five 10 mg tablets of prednisone to cover increased needs for glucocorticoids in emergency situations. This approach has proven effective in the REDUCE trial, where approximately one third of the study patients were discharged from the hospital with biochemically suppressed adrenal glands, but not a single critical incident was provoked by glucocorticoid deficiency during 6 months of follow-up.

6. STUDY DESIGN

6.1 General study design and justification of design

This is an investigator-initiated, placebo-controlled, multicenter noninferiority trial, comparing rapid termination of systemic glucocorticoid treatment with a tapering regime over 4 weeks.

Rationale. To date, there are no published RCTs comparing glucocorticoid tapering with rapid stopping, with regard to hazards resulting from adrenal failure or to disease relapse. Data from trials comparing different tapering schemes in patients treated for inflammatory or autoimmune disorders are scarce and inconclusive. A systematic review found “insufficient evidence about the efficacy and safety of different glucocorticoid withdrawal regimens” and demonstrated “a research gap of high quality randomized trials in a wide range of chronic disorders.”[1] The review evaluated 9 trials (Crohn’s disease: 1; graft versus host disease: 1; bronchial asthma: 5; COPD: 2), 7 of which included less than 50 patients. Our own literature search in PubMed has produced no systematic reviews or meta-analyses published after the report by Richter et al. Given the very small scientific evidence base for the common clinical situation of glucocorticoid withdrawal and the potential for serious untoward effects of these drugs, a noninferiority trial should be conducted to test the hypothesis that an inferior outcome will not result from abrupt glucocorticoid stop even in the presence of biochemically suppressed adrenal glands. From our previous own data, we have no evidence that abrupt withdrawal will endanger patients even in the presence of biochemical adrenal insufficiency. However, it is important to note that potential hazards of adrenal insufficiency will not be underestimated in our trial. Treating physicians will be informed about their patients’ participation in the trial, and patients will be instructed on when and how to use rescue glucocorticoids. These safety measures were also taken in the “REDUCE” trial (cf. paragraph 5.4. above).

Glucocorticoid therapy prior to enrolment. The issue of time and duration of glucocorticoid treatment as well as the daily dosage upon enrolment is critical and has been extensively discussed among the planning committee and the collaborating researchers. In order to maximize the proportion of patients with adrenal suppression, an intuitive approach would be to include participants who are on a current dose as high as possible and have received treatment for as long as possible. However, our own data and published evidence suggest that the cumulative dose and duration of treatment does not allow to predict adrenal suppression[6], although the contrary observation has also been reported.[7] Also, the abrupt stop from a relatively high daily dose (e.g. 30 or 40 mg prednisone-equivalent), administered over a prolonged period (weeks to months) might increase the event rate due to frequent symptoms because of glucocorticoid resistance, even if the HPA axis is recovering. In many inflammatory and autoimmune disorders, glucocorticoids are tapered to prevent a relapse, although tapering schemes are not standardized in rheumatoid arthritis, polymyalgia rheumatica, brain edema due to tumor metastases, sarcoidosis, asthma, and other diseases. Thus, at the time of inclusion, patients must be on a dose low enough to permit an abrupt stop, as judged by the treating physician, but high enough to still suppress the HPA axis. Based on these considerations, the planning committee settled on a treatment duration of ≥ 28 days, and a minimal cumulative dose of 420 mg. The minimum average daily dose must be at least 7.5 mg prednisone-equivalent, and patients must still be on a daily dose of ≥7.5 mg prednisone-equivalent as inclusion criterion. We expect many patients with eligible diseases to have considerably higher cumulative exposure, e.g. those with sarcoidosis, inflammatory bowel disease, polymyalgia rheumatica, rheumatoid arthritis, autoimmune hemolytic anemia, and other autoimmune disorders.

Patients and Procedures. In total, 573 patients will be enrolled. Patients will be randomly assigned in a 1:1 ratio to either prednisone in decreasing doses over 4 weeks or placebo using a centralized secured study website, programmed by the CTU Basel, to ensure allocation concealment. Patients, treating physicians, and study personnel will be blinded to treatment allocation to either prednisone or matching placebo. At inclusion, we will perform a 250 mcg ACTH (Synacthen®) stimulation test ≥24 hrs. after the last glucocorticoid dose to avoid interaction of prednisone with endogenous cortisol. We chose this test because, unlike the low-dose (1 mcg) test, it is widely available and can be performed by personnel of various disciplines. The results of the test will be blinded to treating physicians and investigators, and its value to predict clinical outcome will only be assessed after completion of the trial. As a safety measure, all patients will be instructed about stress coverage as well as signs and symptoms of hypocortisolism and will be provided with emergency medication, as described above (paragraph 5.4). Treating physicians, including those in primary care, will be informed about their patients’ participation in the trial to increase awareness of potentially occurring hypoadrenergic symptoms. Patients will be randomized to the standard (tapering) or the experimental (matching placebo) arm. The rate at which glucocorticoid should be tapered in the standard arm has been extensively discussed among collaborators and other experts from endocrinology and specialties treating prospective study patients (general internists, gastroenterologists, neurologists, pulmonologists). Although ideally chosen for the standard arm in a noninferiority trial, there is no published standard, “best-of-care” glucocorticoid tapering scheme, but a large variety of expert opinions. Also, there are no controlled data indicating the superiority, with respect to clinical outcome of the underlying disease, of one particular tapering scheme over another, or of tapering versus abrupt stopping. Thus, for many inflammatory and autoimmune disorders, there are no specific guidelines as to the tapering regimens. If there are recommendations to taper to a daily dose below 7.5 mg of prednisone equivalent, e.g. in giant cell arteritis, patients with the respective disorder are not eligible for the study. After careful reflection of all these aspects, the planning committee has settled on the regimen depicted in the figure below. Follow-up will be for six months. We do not think that a longer follow-up period is helpful because proportions of patients with suppressed adrenals will be low at six months (2% in our previous REDUCE trial), the necessary sample size does not change appreciably, and loss to follow-up would likely increase.

In order to improve adherence to the study and ensure feasibility, follow-up visits in most study centers will be by telephone only. Visits will be performed early after stopping glucocorticoids in both arms (i.e. at days 7 and 35) to ensure safety; the other two visits will be on days 90 and 180. In a subset of 180 patients, we will also perform clinical visits on days 7 and 35.

Figure: Study Algorithm

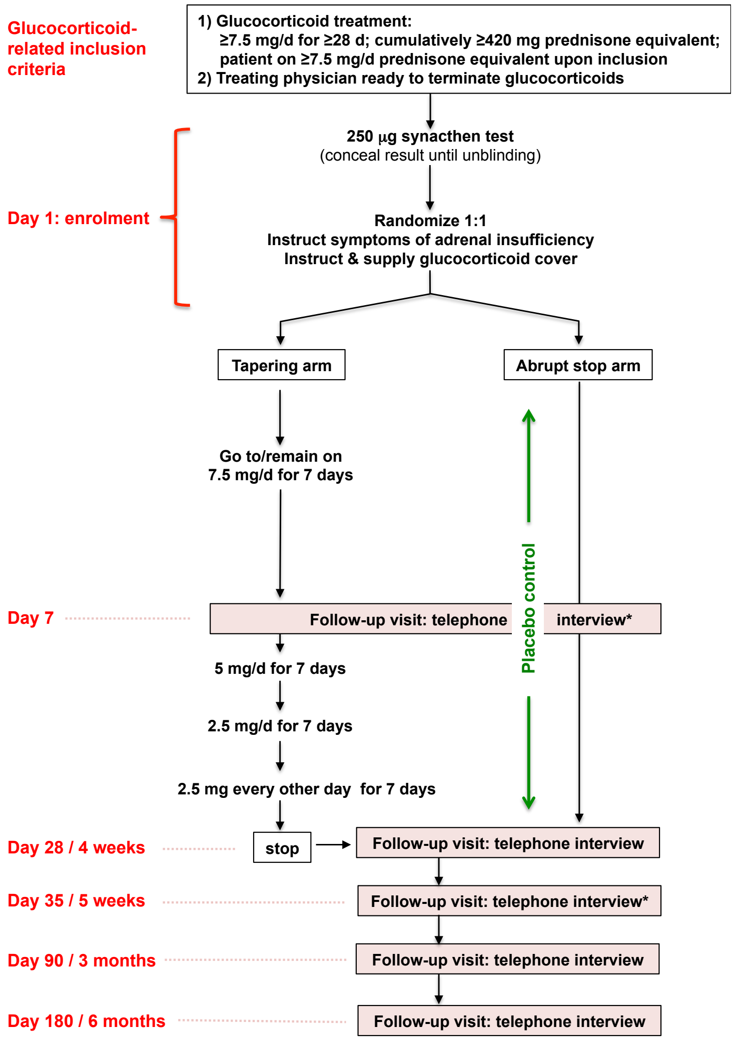

Days 7 and 35 : Due to limited resources, clinical visits will be performed only in a subgroup of 180 patients living in vicinity to their respective trial site, including examination of vital signs and 250 mcg synacthen test.

6.2 Methods of minimising bias

6.2.1 Randomization

The CTU Basel will generate the randomization list, which will be concealed to all study personnel. Patients will be randomly assigned in a 1:1 ratio, stratified according to trial site, age group, and daily prednisone dose at inclusion, to either prednisone in decreasing doses over 4 weeks or placebo. The randomisation procedure will be implemented into the electronic data capture software SecuTrial®. It will include a standard minimisation algorithm which will ensure that the treatment groups are balanced within each stratum. To avoid predictable alternation of treatment allocation, and thus potential loss of allocation concealment, patients will be allocated with a probability of 70 percent to the treatment group that would minimise the difference between the groups within the patient's stratum. Study medication will be pre-packed in glass vials; vial numbers will correspond to the pertinent number on the randomization list.

6.2.2 Blinding procedures

Blinding of trial participants, treating physicians, study doctors, study nurses, outcome assessors and data analysts will be ensured by using identical-looking placebo, packaged in identical vials as the prednisone verum. Vials will be labeled with "Prednisone or Placebo" in an identical fashion.

6.2.3 Other methods of minimizing bias

n.a.

6.3 Unblinding Procedures (Code break)

Unblinding is permissible and procedures for revealing a participant's allocated intervention will be allowed if knowledge of the patient's study medication is essential for further treatment. Unblinding may be performed by the local investigator at any time to ensure patient safety and is executed via the online database tool (SecuTrial). The members of the DSMB have access to the randomization list and have the competence to terminate the trial prematurely.

6.4. End of the trial

The trial end with the last visit of the last patient has been completed (day 180 of the follow-upo). If a SAE is still ongoing at this time point, it will be followed up until its resolution.

7. STUDY POPULATION

7.1 Eligibility criteria

Both in- and outpatients are eligible. Outpatient participants may be followed in specialty clinics or by general practioners in their practice. Inpatient participants may be hospitalized on organ-specific or on general internal medicine wards of the participating trial sites (see p. 12, paragraph 1.2).

Participants fulfilling all of the following <u>inclusion</u> criteria are eligible for the study:

- Informed Consent as documented by signature (Appendix Informed Consent Form)
- Age ≥ 18 years
- Daily glucocorticoid dose ≥ 7.5 mg prednisone-equivalent at the time of inclusion
- Therapy over ≥ 28 days, ≥ 7.5 mg average daily dose, cumulative glucocorticoid dose ≥ 420 mg prednisone-equivalent prior to inclusion
- Tapering not or no longer mandatory to treat underlying disease

The presence of any one of the following <u>exclusion</u> criteria will lead to exclusion of the participant:

- Primary adrenal failure
- Treatment with systemic depot glucocorticoids (e.g. intramuscular, epidural)
- Incapability to administer glucocorticoid cover treatment in situations of stress
- Inability or unwillingness to provide informed consent
- Women who are pregnant or breast feeding,
- Intention to become pregnant during the course of the study,
- Lack of safe contraception, defined as: Female participants of childbearing potential, not using and not willing to continue using a highly effective method of contraception, as defined by the Clinical Trial Facilitation Group (CTFG) for the entire study duration, i.e. hormonal contraception with inhibition of ovulation, intrauterine device, intrauterine hormone-releasing system, bilateral tubal occlusion.
- Known or suspected non-compliance
- Inability to follow the procedures of the study, e.g. due to language problems, psychological disorders, dementia, etc. of the participant,
- Participation in another study with investigational drug within the 30 days preceding and during the present study,
- Previous enrolment into the current study,
- Enrolment of the investigator, his/her family members, employees and other dependent persons
- Untreated hereditary galactose intolerance or lactase deficiency or glucose-galactose malabsorption

If the goals for recruitment are not met, an effort will be made to open additional trial sites.

7.2 Recruitment and screening

Eligible patients are screened for by local investigator or a member of the study team at each center. Participating physicians in private practices will evaluate patients for potential screening and suggest screening for potential participation at one of the trial sites in the Basel area (University Hospital Basel, Kantonsspital Baselland site Liestal,, whichever is most suitable for the patient). Screening is based on analysis of prescribed systemic glucocorticoids, as listed in the patients' charts, and on consultation with the treating physician; this may be done by a study nurse or a study physician. Electronic or paper hospital charts (depending on the prevailing method used by the participating center) will be checked daily. Patients willing to participate will receive written and oral instruction on the trial and give written informed consent.

Patients will not be offered compensation for their participation, except for a refund for their public transportation fees.

7.3 Assignment to study groups

Patients will be randomized by local members of the study teams (physician or trained study nurse) in a 1:1 ratio using an online randomization tool programmed by the CTU Basel which will be accessible over a secured website. The generated randomization code will correspond to the batch number of the pre-packed study medication containing verum or placebo.

7.4 Criteria for withdrawal / discontinuation of participants

The decision about all pharmacological therapy, including unplanned glucocorticoid treatment, will entirely rest with the treating physician, not the study team. Unplanned (rescue) glucocorticoid treatment may also be initiated by the

participating patient in case of symptoms of glucocorticoid deficiency (see also section 8.6. below). The trial compares two regimens with an approved drug in registered indications, thus there are no withdrawal criteria concerning drug-related aspects. Rather, unplanned (rescue) glucocorticoid treatment will be assessed as component of the primary outcome.
Reasons for exclusion of a patient include withdrawal of informed consent or non-compliance. Study subjects may choose to withdraw from the trial, both during the treatment or follow-up phase, without justification. Substitute patients for drop-outs will be recruited. See also point 9.2.5 below.

8. STUDY INTERVENTION

8.1 Identity of Investigational Products (treatment / medical device)

Study drug: prednisone vs. matching placebo.
1. Standard (tapering) arm: patients receive 7.5 mg prednisone/d for 7 days; then 5 mg prednisone/d for 7 days; then 2.5 mg prednisone/d for 7 days; then 2.5 mg prednisone every other day for 7 days, then stop.

2. Intervention (abrupt stop) arm: patients receive matching placebo for 4 weeks, then stop.

8.1.1 Experimental Intervention (treatment)

ICH: Name and description of the investigational product(s).

Placebo:
White placebo tablets containing lactose (P-Tabletten®; diameter 7 mm) will be purchased from Zentiva, Germany. Size, color and divisibility of this product match the 5 mg Prednisone tablet marketed in Switzerland by Galepharm.

8.1.2 Control Intervention (standard/routine/comparator treatment)

Prednisone:

Prednison Galepharm® p.o., white tablets 5 mg each. Can be broken in half.

8.1.3 Packaging, Labelling and Supply (re-supply)

Study drugs will be dispensed in a glass vial labeled according to GMP guidelines, annex 13, in German and French language. Each vial will contain 30 tablets, either verum or placebo.

The identification labels contain the unique Participant Study ID and fulfil the requirements of Annex 13 of the "EUDRALEX Volume 4 - Medicinal Products for Human and Veterinary Use: Good Manufacturing Practice“. Label text will be available in local language of the countries of each participating centre (i.e., German,).

Dosing:
Week 1: Take one and one half tablets, either prednisone (verum) or placebo, daily p.o.
Week 2: Take one tablet, either prednisone (verum) or placebo, daily p.o.
Week 3: Take one half tablet, either prednisone (verum) or placebo, daily p.o.
Week 4: Take one half tablet, either prednisone (verum) or placebo, on days 1, 3, 5, and 7 p.o.

8.1.4 Storage Conditions

The study drug is stored at room temperature (15-25°C) in a locked cabinet, separate from routine stocks.

8.2 Administration of experimental and control interventions

8.2.1 Experimental Intervention

Prednisone and other synthetic glucocorticoids are administered in a variety of inflammatory and autoimmune disorders. For most of these, there are no dose regimens that were established in randomized controlled trials, e.g. by head-to-head comparisons. In this trial, therapy of the underlying disease is entirely at the discretion of the treating physician. This includes the decision that the disease is stable and the glucocorticoid is administered at a daily dose that warrants its termination. Our hypothesis that abrupt glucocorticoid stop will not be inferior to tapering, with respect to clinical outcome, can only be tested in a randomized, placebo-controlled trial. Hence we chose placebo as comparator in the experimental arm.

The experimental intervention consists of oral administration of placebo tablets matching those filled with prednisone for 4 weeks. Follow-up is for 6 months after enrolment.

8.2.2 Control Intervention

The control intervention consists of oral administration of prednisone, tablets in decreasing doses, according to the

following regimen: patients receive 7.5 mg prednisone/d for 7 days; then 5 mg prednisone/d for 7 days; then 2.5 mg prednisone/d for 7 days; then 2.5 mg prednisone every other day for 7 days, then stop.

There is no consensus in published literature on tapering regimes for any specific disease. An expert committee of Swiss endocrinologists therefore convened and recommended the regimen described above. To be eligible for the trial, a patient must be on a daily dose of 7.5 mg prednisone-equivalent or more, because this is the minimum dosage exceeding the endogenous glucocorticoid production, capable of suppressing the hypothalamic-pituitary-adrenal axis. The drug must have been administered systemically, i.e. orally or intravenously, but not in a depot form (intramuscularly, intraarticularly) or topically, since this would preclude the option to taper in defined dosage steps.

Follow-up is for 6 months after enrolment.

8.3 Dose modifications

The decision to start unplanned open-label glucocorticoid therapy during the blinded treatment phase or during follow-up rests with the treating physician or the patient, not the study team . Indication to do so include flare-up of the underlying disease, symptoms and signs of cortisol deficiency such as weakness, apathy, nausea, vomiting, abdominal pain, hyperthermia, hyponatremia, hyperkalemia, hypoglycemia, or adrenal crisis (i.e. glucocorticoid-responsive shock) (see also section 8.6. below). Should the study physician suspect the necessity of open-label glucocorticoid therapy during follow-up, he/she will contact the treating physician to prompt a visit.

8.4 Compliance with study intervention

Patients will be asked to fill out a medication diary over the 4-week treatment phase. Adherence to study medication will be ascertained during the follow-up telephone visits at days 7 and 35 when patients will be asked to count tablets remaining in the vials ("pill count"). All patients will be asked to send back the spare tablets at the end of the treatment phase or return them at the visit on day 35, if this is a hospital visit. A sensitivity analysis will be performed to determine a compliance cutoff, i.e. a critical percentage of ingested study medication, below which an association with the outcome is visible.

8.5 Data Collection and Follow-up for withdrawn participants

Patients who choose to withdraw from the study during the treatment intervention will be considered as drop-outs. Patients who choose to withdraw from the study during the follow-up period (i.e. refuse to participate in the telephone interviews on days 35, 90, and 180 or to come to the outpatient visit on day 35, if applicable) will be asked for permission to contact their treating physician for inquiry of the 1° outcome and to use their data collected so far. This will prevent their drop-out. If patients do not permit the inquiry of the 1° outcome, their data will be considered right-censored from the last documented contact onward.

8.6 Trial specific preventive measures

Each study participant will be given five 10 mg tablets of prednisone for open label/rescue therapy in case of urgent need for glucocorticoid administration. Each study participant will be instructed about the signs and symptoms of glucocorticoid deficiency upon enrolment and will receive a written document specifying these signs and symptoms in a well understandable manner (see screen 6.11, "Stressprophylaxe").
Open label or rescue glucocorticoid therapy will be recorded on the CRF. Its impact on the study is such that unplanned glucocorticoid therapy is one of the components of the primary, as well as one of the secondary outcomes.

8.7 Concomitant Interventions (treatments)

Any concomitant care or/and intervention is at the discretion of the treating physician and will be recorded on the CRF. If such care/intervention leads to hospitalization, it will affect the trial as component of the primary as well as one of the secondary outcomes. Any concomitant care and interventions will be recorded in the CRF.

8.8 Study Drug Accountability

The study drugs are shipped to the individual trial sites by the pharmacy of the University Hospital Basel, which keeps the log for each of the numbered packages containing the study medication for one participant. Prednisone and placebo can be shipped at ambient temperature (min. 15°C, max. 25°C). The local stud nurse will accept the delivery and store the study medication at room temperature in a locked cabinet, separate from routine stocks.

The Study Medication provided for this study will only be dispensed to enrolled Study Participants and used only

as directed in the study protocol. The local Principal Investigators at each Study Site are responsible for all supplies of Study Medication from the time of receipt until destruction of any unused or non-dispensed Study Medication. For this purpose, the Investigator or delegate will keep an accurate Study Medication accountability record and destruction log. At the end of the study, one copy of the Study Medication Accountability record will be sent to the sponsor-investigator and one kept in the Investigator's Site file.

8.9 Return or Destruction of Study Drug

Unused study drugs and packing material will be destroyed by the local pharmacy of each trial site. A certificate thereof will be delivered.

9. STUDY ASSESSMENTS

9.1 Study flow chart(s) / table of study procedures and assessments

Study Periods	Screening	Treatment, Intervention Period			Follow-up		
Visit	1	2	3	4	5	6	7 (final)
Time (hour, day, week)	-7d to -1d	Day 1	Day 7 ±1d	Day 28 ±3d	Day 35 ±3d	Day 90 ± 5d	Day 180 ±7d
Patient Information and Informed Consent	x						
In- /Exclusion Criteria	x						
Vital Signs (heart rate, blood pressure), body weight, height [only day 1])		x	x **		x **		
Laboratory Tests: 250 mcg Synacthen® test		x	x **		x **		
Pregnancy test in menstruating women not taking oral contraceptive	x						
Randomisation		x					
Interview Assessment: Primary outcome variables*† - fill out CRF			x	x	x	x	x
Interview Assessment: Secondary outcome variables*†- fill out CRF			x	x	x	x	x
Interview Assessment:*†: symptoms & signs of hypocortisolism; concomitant therapy; serious adverse events; therapeutic interventions by other physicians – fill out CRF		x	x	x	x	x	x
Dispense and start study medication		x					
Terminate study medication				x			
Return surplus study medication					x††		

* Assessed in all study participants
** Due to restricted resources, assessed only in a subgroup of 180 patients living near their respective trial site.
† Assessed by telephone, except day 1 (all centers) and days 7 and 35 (subgroup of 180 patients that have 2 clinical visits during follow-up). If desired by a trial site, follow-up telephone interviews can be performed centrally by a study coordinator. In these instances, ICF shall be adapted accordingly for such site(s).
†† by mail, except if a clinical visit is performed on day 35.

9.2 Assessments of outcomes

9.2.1 Assessment of primary outcome

Assessment tools of 1° outcome measures:

- Questionnaire: Hospitalization? Unplanned glucocorticoid therapy? Adrenal crisis?
- Treating physician interview: Death?

The 1° outcome can be assessed during a telephone interview with the patient using a questionnaire.
If a patient cannot be reached, the treating physician will be contacted to inquire about the possibility of the patient's death.

The 1° outcome will be assessed by telephone on days 7, 28, 35, 90, and 180; ± the variations indicated in the study flow chart. Additionally, a subgroup of 180 patients living near their respective trial site will be seen for a clinical visit on study days 7 and 35. These clinical visits include vital signs (heart rate, blood pressure in recumbent and upright positions), and a 250 mcg Synacthen® test.

9.2.2 Assessment of secondary outcomes

The secondary outcomes will be assessed using telephone interviews with patients. Questionnaires will be used to assess outcomes, as described above (see 9.2.) Treating physicians will be contacted by telephone to inquire about the secondary outcomes, cumulative systemic overall glucocorticoid dose, cumulative systemic glucocorticoid dose to treat or prevent adrenal failure, cumulative systemic glucocorticoid dose to treat relapse of underlying disease; as well as the primary outcome component of death, where applicable.

Assessment tools of 2° outcome measures:

- Questionnaire: individual components of 1° outcome; SF-12 survey; symptoms of hypocortisolism
- Signs of hypocortisolism, in subgroup as specified in above table: measure blood pressure (supine and standing), measure heart rate.
- Treating physician interview: cumulative systemic overall glucocorticoid dose? Cumulative systemic glucocorticoid dose to treat or prevent adrenal failure? Cumulative systemic glucocorticoid dose to treat relapse of underlying disease?
- Perform 250 mcg ACTH (Synacthen®) test*: in subgroup as specified in the above table.
- Measure HbA1c value: in subgroup as specified in the above table.
- Determine length of hospital stay (if hospitalized upon study inclusion): Study hospital chart

*The 250 mcg ACTH (Synacthen®) test is a routine procedure for the assessment of the functional status of the hypothalamus-pituitary-adrenal axis. The test is performed as follows. It can be done at any time during the day without the need of a fasting state. A blood sample is drawn for measurement of baseline serum cortisol. 1 ampoule (250 mcg) of tetracosactide (beta-1-24 – corticotropin; Synacthen®) is injected intravenously. 30 and 60 minutes after the injection, blood samples are drawn for measurement of stimulated serum cortisol.

The 2° outcomes will be assessed by telephone on days 7, 28, 35, 90, and 180. Additionally, a subgroup of 180 patients will be seen for a clinical visit on study days 7 and 35. These clinical visits include vital signs (heart rate, blood pressure while in recumbent and upright position, and a 250 mcg Synacthen® test.

9.2.3 Assessment of other outcomes of interest

n.a.

9.2.4 Assessment of safety outcomes

Safety parameters include the components of the primary outcome: Time to first occurrence of hospitalization; death, initiation of unplanned systemic glucocorticoid therapy, or adrenal crisis.

Safety outcomes will be assessed by telephone on days 7, 28, 35, 90, and 180. Additionally, a subgroup of 180 patients will be seen for a clinical visit on study days 7 and 35. These clinical visits include vital signs (heart rate, blood pressure in recumbent and upright position), and a 250 mcg Synacthen® test.

9.2.4.1 Prednisone side effects

Prednisone side effects are documented as Adverse Events (AEs). According to the official Swiss drug registry published by (www.swissmedicinfo.ch), these include the following: infection, allergic reactions, arterial hypertension, hyperglycemia, psychic symptoms (mood changes, depression, productive symptoms), arterial hypertension, gastrointestinal ulceration, osteoporosis, aseptic bone necrosis, nausea, weight gain, atrophic changes or easy bruising of the skin, erythema, muscle weakness.

These side effects will be recorded as “present” if patients report new or intensified disease-specific pharmacological therapy initiated by their treating physicians or other medical personnel (in the case of infection, allergic reactions, arterial hypertension, hyperglycemia, psychic symptoms, gastrointestinal ulceration, osteoporosis, aseptic bone necrosis), or if patients report them during the interview (in the case of nausea, weight gain, atrophic changes or easy bruising of the skin, erythema, muscle weakness).

Prednisone side effects will be assessed upon enrolment and during each of the visits during the intervention follow-up period (telephone interview or clinical visit, at the time points specified above (please see 9.1., 9.2.1, 9.2.2). They will be recorded on the CRF.

9.2.4.2 Laboratory parameters

In menstruating females willing to participate in the trial, a urine pregnancy test will be performed at the screening

visit.
In all trial participants, a 250 mcg Synacthen® test will be performed on study day 1. Results will be concealed until the end of the trial.

In all trial participants, the serum remaining from the basal cortisol measurement will be frozen at -20°C as reserve for later batch analyses.

In a subgroup of 180 patients the 250 mcg synacthen test as well as the storage of 5 ml reserve serum will be repeated on study days 7 and 35.

9.2.4.3 Vital signs

Blood pressure and heart rate in lying and supine position after at least 5 minutes resting will be measured in all participating patients on study day 1. The same measurements will be repeated in a subgroup of 180 patients on days 7 and 35.

9.2.5 Assessments in participants who prematurely stop the study

Patients who choose to withdraw from the study during the treatment intervention will be considered as drop-outs. Patients who choose to withdraw from the study during the follow-up period (i.e. refuse to participate in the telephone interviews on days 35, 90, and 180 or to come to the outpatient visit on day 35, if applicable) will be asked for permission to contact their treating physician for inquiry of the 1° outcome and to use their data collected so far. This will prevent their drop-out. If patients do not permit the inquiry of the 1° outcome, their data will be considered right-censored from the last documented contact onward.

9.3 Procedures at each visit

9.3.1 Screening visit (visit 1)

Evaluate for eligibility according to inclusion and exclusion criteria. Obtain informed consent. Measure height and weight. Perform urine pregnancy test if appropriate (in menstruating women). Schedule visit 2.

9.3.2 Day 1 (visit 2)

Measure heart rate and blood pressure in recumbent and supine position after at least 5 minutes resting. Randomize patient. Perform 250 mcg Synacthen® test. Draw blood for reserve serum. For reserve serum: centrifuge sample, pipet serum into 1 ml aliquots, store at -20°C. Assess VAS for general health status. Fill out CRF. Dispense trial medication. Schedule visit 3.

9.3.3 Day 7 ± 1d (visit 3)

A. Telephone interview: Assess VAS for general health status. Fill out CRF. Ask patient about pill count. Schedule visit 3.
B. Clinical visit (subgroup of 180 patients living near their respective trial site): Measure heart rate and blood pressure in recumbent and supine position after at least 5 minutes resting. Perform 250 mcg Synacthen test. Draw blood for reserve serum. For reserve serum: centrifuge sample, pipet serum into 1 ml aliquots, store at -20°C. Schedule visit 4.

9.3.4 Day 28 ± 3d (visit 4)
Telephone interview: Assess VAS for general health status. Fill out CRF. Ask patient about pill count at termination of trial medication, ask patient to send back spare study tablets or bring them along if a clinical visit is planned on day 35. Schedule visit 5.

9.3.5 Day 35 ± 3d (visit 5)
A. Telephone interview: Assess VAS for general health status. Fill out CRF. Schedule visit 6.
B. Clinical visit (subgroup of 180 patients living near their respective trial site): Measure heart rate and blood pressure in recumbent and supine position after at least 5 minutes resting. Perform 250 mcg Synacthen test .Draw blood for reserve serum. For reserve serum: centrifuge sample, pipet serum into 1 ml aliquots, store at -20°C. Schedule visit 6.

9.3.6 Day 90 ± 5d (visit 6)
Telephone interview: Assess VAS for general health status. Fill out CRF.

9.3.7 Closeout visit Day 180 ± 7d (visit 7)
Telephone interview: Assess VAS for general health status. Fill out CRF.

10. SAFETY

10.1 Drug studies

During the entire duration of the study, all serious adverse events (SAEs) are collected, fully investigated and documented in source documents and case report forms (CRF). Study duration encompasses the time from when the participant signs the informed consent until the last protocol-specific procedure has been completed, including the follow-up period of six months.

10.1.1 Definition and assessment of serious adverse events and other safety related events

A Serious Adverse Event (SAE) is defined as any of the following:
Hospitalization, death, life-threatening incident, persistent disability, or other medically important condition.

Both Investigator and Sponsor-investigator make a causality assessment of the event to the study drug, based on the criteria listed in the ICH E2A guidelines:

Relationship	Description
Definitely	Temporal relationship<br>Improvement after dechallenge*<br>Recurrence after rechallenge<br>(or other proof of drug cause)
Probably	Temporal relationship<br>Improvement after dechallenge<br>No other cause evident
Possibly	Temporal relationship<br>Other cause possible
Unlikely	Any assessable reaction that does not fulfil the above conditions
Not related	Causal relationship can be ruled out
*Improvement after dechallenge only taken into consideration, if applicable to reaction	

In agreement with the new law on clinical research (ordinance ClinO), “adverse events” are not documented; however, potential side effects of glucocorticoids (cf. section 9.2.4.1 above) are assessed and recorded on the CRF.

Suspected Unexpected Serious Adverse Reactions (SUSARs)

The Sponsor-Investigator (Chief Investigator) evaluates any SAE that has been reported regarding seriousness, causality and expectedness. If the event is related to the investigational product and is both serious and unexpected, it is classified as a SUSAR.

10.1.2 Reporting and documentation of Serious Adverse Events (SAE), Adverse Events (AEs), and other safety related events

All SAEs are reported by the local investigator via the electronic form supplied in SecuTrial to the Sponsor-Investigator of the study immediately after obtaining knowledge. The Sponsor-Investigator automatically receives an immediate notification by E-mail after the SAE form has been completed electronically. He will evaluate the SAE and discuss it with the local staff if deemed necessary. The electronic SAE form may then be amended by the local team, which will create another automatic E-mail to the Sponsor-Investigator. Ongoing SAEs are followed up and reported via SecuTrial until their end.
In addition to the electronic documentation via SecuTrial, all SAEs are documented in paper form, signed by the local staff, and filed in the Trial Site File.
The Sponsor-Investigator (Chief Investigator) is responsible for informing all competent authorities (CA) and ethical committees (CEC) of the concerned Member States of any Serious Adverse Events as per local requirements-

SAEs resulting in death are reported to the local Ethics Committee in Switzerland and Germany within 7 days. The other Ethics Committees involved in the trial receive SAEs resulting in death in Switzerland via Sponsor-Investigator within 7 days.

Documenting of AEs
These are documented in SecuTrial as prednisone side effects and include the following: infection, allergic reactions, arterial hypertension, hyperglycemia, psychic symptoms (mood changes, depression, productive symptoms), arterial hypertension, gastrointestinal ulceration, osteoporosis, aseptic bone necrosis, nausea, weight gain, atrophic changes or easy bruising of the skin, erythema, muscle weakness. Additional AE information may be documented in a free text field in SecuTrial.

Reporting of SUSARs

All SUSARs are reported by the local investigator via the electronic form supplied in SecuTrial to the Sponsor-

Investigator immediately after obtaining knowledge. The Sponsor-Investigator will report all SUSARs in compliance with applicable laws and regulations to all Competent Authorities of the concerned Member States (e.g. the BfArM in Germany, Swissmedic in Switzerland, and CECs of all study sites (lead CEC in Switzerland) immediately after obtaining knowledge.

In addition, the Sponsor-Investigator (Chief Investigator) must inform all local Investigators participating in the clinical study of the occurrence of a SUSAR.

Reporting of Safety Signals
n.a.

Reporting and Handling of Pregnancies
n.a.

Periodic reporting of safety

An annual safety report is submitted once a year to the Competent Authorities and CECs of all study sites (lead CEC in Switzerland and Germany)- by the Sponsor-Investigator.

10.1.3 Follow up of Serious Adverse Events

Participants with an SAE will be followed up until the SAE has resolved or the patient is lost to follow-up. If a patient with an SAE wishes to leave the study prematurely, he/she will be asked permission to assess the primary outcome parameters by obtaining the information from the treating physician. If a participant is lost to follow-up but has not expressed the wish to leave the study prematurely, the treating physician will be contacted to assess the primary outcome.

If a participant has an SAE ongoing beyond the regular time point of study termination, i.e. day 180, the SAE will nevertheless be followed until its resolution (or the loss of follow-up), including the cumulative the dose of prednisone-equivalent administered to treat the event.

11. STATISTICAL METHODS

11.1 Hypothesis

The aim of the trial is to test the hypothesis that rapid termination of glucocorticoid treatment (experimental arm) is noninferior to the tapering regime (control arm) with regard to the time to event (primary composite outcome). Accordingly, the null hypothesis corresponds to inferiority of the abrupt glucocorticoid tapering regime.

11.2 Determination of Sample Size

To assess the primary outcome measure, a Cox proportional hazards regression model will be fitted to the data. Non-inferiority will concluded if the two-sided 95% confidence interval of the hazard ratio between the experimental and the control arm lies entirely below the critical hazard ratio defined as

$$HR = \frac{\lambda_e}{\lambda_c} = \frac{\frac{-\log(\pi_{et})}{t}}{\frac{-\log(\pi_{ct})}{t}} = \frac{\log(\pi_{e_t})}{\log(\pi_{c_t})} = \frac{\log(\pi_{c_t} - m)}{\log(\pi_{c_t})}$$

where λ_e and λ_c are the hazard rates in the experimental and control arms, t is a fixed point of time, π_{et} and π_{ct} are the proportions of event-free patients at time t, and m is the non-inferiority margin expressed as the additional proportion of patients having had an event in the experimental arm, assuming that the occurrence of events follows an exponential distribution. Noninferiority will be concluded if the upper limit of the two-sided 95% confidence interval of the hazard ratio lies below the critical hazard ratio.

For the sample size estimation, the following assumptions were made:

- Patients are randomized to the control and experimental arms in a 1:1 ratio.
- After inclusion, all patients are followed for 6 months. Patients without an event within 6 months are considered right-censored and are not followed any further even though the trial might be ongoing.
- 10% of patients drop out within the 6 months follow-up period. The drop-out events are distributed uniformly over this period.
- The event rate within the 6 months follow-up period, i.e., the proportion of patients with an event, is 40%, both for the control and the experimental arms. We estimated this overall figure based on expected relapse rates for the various eligible diseases, as indicated by clinical experts and published data, e.g. in COPD or inflammatory bowel disease. Our estimation is conservative since the other components of the combined primary outcome will contribute to the event rate.
- The survival curves in the control and experimental arms follow the same exponential distribution. The rate parameter of the exponential distribution is chosen so that the corresponding cumulative distribution function value at 6 months equals the assumed event rate.
- The non-inferiority margin is defined as an absolute increase of the event rate by 13% over 6 months. We used a modified Delphi approach to determine this margin. 10 experienced clinicians board-certified in internal medicine and/or rheumatology, gastroenterology, and endocrinology were individually asked to define the maximum increase in outcome events they would accept for the benefit of rapid glucocorticoid stop and gain of high-quality scientific data in the area of steroid withdrawal. Assuming an event rate of 40% in the standard treatment arm, the mean tolerable increase under experimental treatment was 13.6% (range, 5 to 20%; median, 13%).
- The significance level is 5%.

For each combination of the noninferiority margins 7.5%, 7.6%, …, 17.5%, and the sample sizes n = 200, 210, …, 1200, 999 individual data sets were simulated based on the above assumptions. A Cox proportional hazards regression model was fitted to each data set, and the hazard ratios for the experimental *vs.* the control arm with the associated 95% confidence interval were estimated. Noninferiority was concluded if the upper limit of the two-sided 95% confidence interval of the hazard ratio was below the critical hazard ratio (see above equation). The power was estimated as the proportion of positive conclusions divided by the total number of the 999 iterations per parameter combination. To achieve a power of 80%, 573 patients (95% CI, 565 to 580) need to be included, assuming an event rate of 40% over the 6 months follow-up period and setting the non-inferiority margin to 13% (Figure). The critical hazard ratio for this noninferiority scenario is 1.478.

The sample size of n=573 is a conservative estimation, since the assumed drop-out rate of 10% is relatively high.

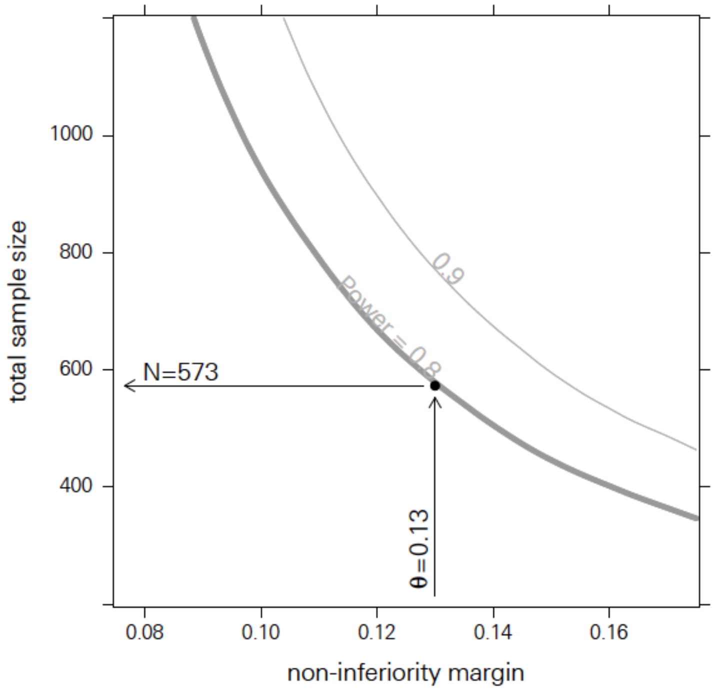

11.3 Statistical criteria of termination of trial

NA

11.4 Planned Analyses

Detailed methodology for summaries and statistical analyses of the data collected in this trial will be documented in a statistical analysis plan. The statistical analysis plan will be finalised before database closure and will be under version control at the Clinical Trial Unit, University Hospital Basel. Deviations from the analyses described below will be listed in a separate section of the analysis plan.

11.4.1 Datasets to be analysed, analysis populations

The full analysis set (FAS) consists of all patients who are randomised.
The intention-to-treat (ITT) set is a subset of the FAS that consists of all patients for whom the primary endpoint is available. Patients are assigned to the treatment arm they are randomised to.
The per protocol (PP) set includes the same patients as the ITT set, but patients are assigned to the treatment arm that corresponds to the actually obtained treatment.

11.4.2 Primary Analysis

A Cox proportional hazards regression model with time to the (combined) primary endpoint as the response and treatment arm as the explanatory variable will be fitted, stratified after study centre, prednisone dose, and age group. Non-inferiority will be concluded if the two-sided 95% confidence interval of the hazard ratio (HR) between the experimental and the control arm lies entirely below the critical HR ratio of 1.478. The analysis will be conducted on the PP set.

11.4.3 Secondary Analyses

Secondary outcomes:

Performance of the 250 mcg corticotropin stimulation test. If this test is clinically relevant, it is assumed that in patients with a normal test result, the two study arms will not differ with regard to the primary outcome, whereas patients with a pathological test result will experience an event more often after the rapid termination (experimental arm) than after tapering (control arm). It is thus hypothesized that there is an interaction between the test result and the glucocorticoid withdrawal scheme. To test for a significant interaction, the Cox proportional hazard regression model used to assess the primary objective is extended by the stimulation test result (passed vs. failed) and its interaction with the treatment group (rapid vs. tapering). If a significant interaction is found, subgroup analyses are performed for the two treatment groups in order to estimate the strength of the association between test result and primary outcome.

The time to first occurrence of individual components of the primary outcome is, in analogy to the (composite) primary outcome, also analysed with Cox proportional hazards regression models.

Cumulative overall systemic glucocorticoid dose, cumulative systemic glucocorticoid dose administered to treat or prevent adrenal failure, cumulative systemic glucocorticoid dose administered to treat relapse of disease (specified for each disease), general health status as self-assessed by the patients on a VAS, blood pressure (supine and standing), hospital length of stay, weakness, hypothermia, nausea, vomiting, abdominal pain, and fatigue are analysed with linear regression models.
In all analyses, the variables for which randomisation was stratified (center and age group) are included as covariates for adjustment. If model assumptions are not met, transformations of outcome variables and the use of generalised linear (instead of linear) models are considered.

Besides the secondary outcomes, potential glucocorticoid-induced side effects will be assessed and documented on the CRF. Proportions of side effects will be compared between treatment groups and analyzed using logistic regression.

Subgroup analyses:
Pre-specified subgroups defined by the following parameters will be analyzed by the trial statistician:

1. Daily glucocorticoid dose (in mg prednisone-equivalent) at the time of inclusion
2. Cumulative glucocorticoid dose (in mg prednisone-equivalent) in the 28 days prior to inclusion
3. Sex
4. Concomitant immunosuppressive drugs at inclusion or during follow-up
5. Underlying disorder
6. Result of the 250 mcg synacthen® test

11.4.4 Interim analyses

Once 70% of the planned sample size will have their primary endpoint assessed, we will perform a blinded, pooled, sample size re-estimation . To this end, the global rate of events – pooled over both treatment arms – will be calculated, and compared to the assumed rate in the sample size estimation. The sample size estimation will be repeated as performed originally and the study's sample size adjusted accordingly in case more patients are needed. In no case will the originally calculated sample size be reduced.

11.4.5 Safety analysis

Two types of safety analyses will be performed: periodic analyses for the DSMB's meetings, and a final safety analysis at the end of the trial.
Safety analyses for DSMB meetings will summarize the rates of safety outcomes (as defined above, section 10) pooled over both treatment arms. Event types and relevance as defined for SAEs will be included in the report. The DSMB has the right to request an analysis stratified by treatment arm, or completely unblinded in case of concern for patient safety. Safety analysis for the DSMB is performed by the independent DSMB statistician.
The final safety analysis for the trial will summarize the rates of safety outcomes stratified by treatment arm and will be performed by the trial statistician after study completion.

11.4.6 Deviation(s) from the original statistical plan

If substantial deviations of the analyses as outlined in these sections are needed for whatever reason, the protocol will be amended. All deviations from the protocol or from the detailed statistical analysis plan will be listed and justified in a separate section of the final statistical report.

11.5 Handling of missing data and drop-outs

We will strive to make a complete case analysis, which will likely be feasible because the primary outcome is composed of clearly defined clinical events that can easily be tracked and inquired from the treating physicians if necessary. If a substantial proportion of patients have missing data, we will consider the use of multiple imputation.
Patients who leave the trial during the blinded treatment phase are replaced only if the number of total drop-outs is >10%.

12. QUALITY ASSURANCE AND CONTROL

The Sponsor will be responsible for implementing and maintaining quality assurance and quality control systems with written SOPs and Working Instructions at all participating trial sites. The PI is responsible for proper training of all involved study personnel.

12.1 Data handling and record keeping / archiving

The data will be entered either manually or directly online (e.g. eCRF) into the SecuTrial© data base of the CTU Basel. The data are stored on a dedicated server at the University Hospital Basel. Investigator site files will be archived at each local trial site. Trial master file will be archived at the sponsor/investigator's site.

12.1.1 Case Report Forms

For each study participant an eCRF will be generated and maintained on the SecuTrial© data base. To facilitate data entry, source documentation files will be generated. CRFs will be kept current to reflect subject status at each phase during the course of study. Participants will not be identified in the CRF by name or initials and birth date. Appropriate coded identification, e.g. participant number incombination with year of birth will be be used.

Each study team member will have their own personal login access for the eCRF and data base, with specific rights described in the function delegation log.

12.1.2 Specification of source documents

Source data will be available at the site to document the existence of the study participants. Source data may include original documents (including electronic patient records) relating to the study, the medical treatment or medical history of the participant. If such records are not available at a follow-up visit, e.g. if a patient was not hospitalized or seen by a physician since the last visit, pertinent data regarding the 2° outcome measures may be entered directly into the eCRF on the SecuTrial data base.

The Informed Consent Forms are additional source documents.

12.1.3 Record keeping / archiving

All study data will be archived for a minimum 10 years after study termination or premature termination of the clinical trial.

12.2 Data management

12.2.1 Data Management System

The clinical trial data will be collected in an electronic data capture (EDC) system, named secuTrial®. The EDC system runs on a server maintained by the IT-department of the University Hospital Basel. The electronic CRF (eCRF) is implemented (set-up and adjusted) by the data management group at the Clinical Trial Unit (CTU) at the University Hospital Basel.

Each study center (site) is responsible for data entry into the EDC system.

12.2.2 Data security, access and back-up

The EDC system is accessible via a standard browser on a WWW-connected device. Password protection ensures that only authorized persons can enter the system to view, add or edit data according to their permissions. User administration and user training is performed by the CTU according to predefined processes. Back-up of secuTrial® study data is performed according to the processes of the IT-department of the University Hospital Basel.

12.2.3 Analysis and archiving

The EDC will be locked after all data was monitored and all raised queries have been resolved. Data is exported and transferred to the investigator by the CTU according to internally defined processes. Data will be archived by the investigator.

12.2.4 Electronic and central data validation

Data is entered into the eCRF and can be validated for completeness and discrepancies automatically. An audit trail system maintains a record of initial entries and changes (reasons for changes, time and date of changes, user identification of entry and changes).

At all times, the Investigator has final responsibility for the accuracy and authenticity of all clinical data.

The data entered into the eCRF will be reviewed by the responsible investigator and an independent monitor will raise queries using the query management system implemented. Designated investigators have to respond to the query and confirm or correct the corresponding data. Thereafter the monitor can close the query.

12.3 Monitoring

Monitoring will be outsourced to the Clinical Trial Unit Basel, except in select trial sites which will be monitored by other institutions (see paragraph 1.5). Monitoring will be performed on the basis of the monitoring plan accepted by the sponsor. Monitoring includes a site initiation visit (for each individual site) prior to the enrolment of the first participant per site. According to the monitoring plan there will be routine monitoring visits during the trial and a close-out visit at each site after last patient-last visit. The monitoring includes e.g. ICFs, safety data and patient selection criteria.
The source data/documents will be accessible to monitors and questions will be answered during monitoring.

12.4 Audits and Inspections

The study documentation and the source documents will be accessible to auditors/inspectors (also CEC and CA) and questions will be answered during inspections. All involved parties will keep the participant data strictly confidential.

12.5 Confidentiality, Data Protection

Direct access to source documents will be permitted for purposes of monitoring (12.3), audits and inspections (12.4).

Access to protocol, dataset, statistical code will be granted to CEC, CA, the DSMB during and after the study.

Project data will be handled with uttermost discretion and is only accessible to authorized personnel who require the data to fulfil their duties within the scope of the research project. On the CRFs and other project specific documents, participants are only identified by a unique participant number.

12.6 Storage of biological material and related health data

Serum samples will be stored at -20°C for maximally 5 years after the last patient has completed the study.
Storage is allowed only with the participants’ consent, independent of the study.

13. PUBLICATION AND DISSEMINATION POLICY

The trial design will be registered at ISRCTN and thus be made accessible to the public. Trial results will be submitted to an international peer-reviewed journal. We do not intend to use professional writers to prepare the manuscript.

14. FUNDING AND SUPPORT

14.1 Funding

This trial is supported by the Swiss National Science Foundation (grant no. 32003B_163133), by the Hemmi-Stiftung, the Gebauer-Stiftung, the Huof und Elsa Isler-Fonds, and by funds from Kantonsspital Baselland. Applications for further funding by other agencies are pending.

14.2 Other Support

Intellectual and infrastructural support has been/will be received from the principle investigators at the local trial sites. The trial is supported by the Medical Faculty, University of Basel.

15. INSURANCE

Insurance is provided by the sponsor. A copy of the certificate is filed in each investigator site file and the trial master file. Documentation of guarantee of liability is provided in separate document.

16. REFERENCES

16.1. References as cited in the text:

1. Richter B. et al. Endocrinol Metab Clin North Am 2002; 31:751-78.
2. Leuppi J.et al., JAMA 2013; 309, 2223–31
3. Schütz P. et al., Eur J Endocrinol 2015; 173:19-27
4. Jasani M.K. et al., Acta rheumatol Scand1968; 14:65-70
5. Kehlet H., Binder C., Br Med J 1973; 2: 147-9
6. Schlaghecke R. et al., N Engl J Med 1992; 326(4): 226-30
7. Sacre K et al., J Clin Endocrinol Metab 2013; 98(8): 3199-3205
8. Christy N.P., N Engl J Med. 1992;326(4):266–267)
9. Sampson P.A. et al., Lancet 1961;1(7191):1377

16.2. General sources applying to this study protocol and referred to in the text as appropriate:

- Declaration of Helsinki, Version October 2013, (http://www.wma.net/en/30publications/10policies/b3/index.html)
- International Conference on Harmonization (ICH, 1996) E6 Guideline for Good Clinical Practice. (http://www.ich.org/fileadmin/Public_Web_Site/ICH_Products/Guidelines/Efficacy/E6_R1/Step4/E6_R1__Guideline.pdf)
- International Conference on Harmonization (ICH, 1997) E8 Guideline: General Considerations for Clinical Trials http://www.ich.org/fileadmin/Public_Web_Site/ICH_Products/Guidelines/Efficacy/E8/Step4/E8_Guideline.pdf)
- Humanforschungsgesetz, HFG Bundesgesetz über die Forschung am Menschen (Bundesgesetz über die Forschung am Menschen, HFG) vom 30. September 2011/ Loi fédérale relative à la recherche sur l'être humain (loi relative à la recherche sur l'être humain, LRH) du 30 septembre 2011. (http://www.bag.admin.ch/themen/medizin/00701/00702/07558/index.html?lang=de)
- Verordnung über klinische Versuche in der Humanforschung (Verordnung über klinische Versuche, KlinV) vom 20. September 2013 / Ordonnance sur les essais cliniques dans le cadre de la recherche sur l'être humain (Ordonnance sur les essais cliniques, OClin) du 20 septembre 2013. (http://www.bag.admin.ch/themen/medizin/00701/00702/12310/index.html?lang=de)
- Heilmittelgesetz, HMG Bundesgesetz über Arzneimittel und Medizinprodukte (Heilmittelgesetz, HMG) vom 15. Dezember 2000/Loi fédérale sur les médicaments et les dispositifs médicaux (Loi sur les produits thérapeutiques, LPT) du 15 décembre 2000. (http://www.admin.ch/ch/d/sr/8/812.21.de.pdf)
- ISO 14155:2011 Clinical investigation of medical devices for human subjects -- Good clinical practice (www.iso.org)
- ISO 10993 Biological evaluation of medical devices (www.iso.org)
- WHO, International Clinical Trials Registry Platform (ICTRP) (http://www.who.int/ictrp/en/)

17. APPENDICES

Case Report Form (CRF), Patient Information and Informed Consent Form (ICF), as well as participant information sheets about glucocorticoid emergency cover treatment (Stressprophylaxe and Notfallausweis) are uploaded on the pertinent screens of the Basec platform.
